# FSP1-mediated lipid droplet quality control prevents neutral lipid peroxidation and ferroptosis

**DOI:** 10.1038/s41556-025-01790-y

**Published:** 2025-10-29

**Authors:** Mike Lange, Michele Wölk, Vivian Wen Li, Cody E. Doubravsky, Joseph M. Hendricks, Shunji Kato, Yurika Otoki, Benjamin Styler, Sean L. Johnson, Cynthia A. Harris, Kiyotaka Nakagawa, Isabel F. Snodgrass, Dohee Kim, John W. Newman, Maria Fedorova, James A. Olzmann

**Affiliations:** 1 Department of Molecular and Cell Biology, University of California, Berkeley, Berkeley, CA USA; 2 Department of Nutritional Sciences and Toxicology, University of California, Berkeley, Berkeley, CA USA; 3 Center of Membrane Biochemistry and Lipid Research, University Hospital and Faculty of Medicine Carl Gustav Carus of TU Dresden, Dresden, Germany; 4 Laboratory of Food Function Analysis, Graduate School of Agricultural Science, Tohoku University, Sendai, Japan; 5 West Coast Metabolomics Center, Genome Center, University of California, Davis, Davis, CA USA; 6 United States Department of Agriculture, Agricultural Research Service, Western Human Nutrition Research Center, Davis, CA USA; 7 Department of Nutrition, University of California, Davis, Davis, CA USA

**Keywords:** Organelles, Fats, Lipidomics

## Abstract

Lipid droplets (LDs) are organelles that store and supply lipids, based on cellular needs. Although mechanisms preventing oxidative damage to membrane phospholipids are established, the vulnerability of LD neutral lipids to peroxidation and protective mechanisms are unknown. Here we identify LD-localized ferroptosis suppressor protein 1 (FSP1) as a critical regulator that prevents neutral lipid peroxidation by recycling coenzyme Q10 (CoQ10) to its lipophilic antioxidant form. Lipidomics reveal that FSP1 loss leads to the accumulation of oxidized triacylglycerols and cholesteryl esters, and biochemical reconstitution of FSP1 with CoQ10 and NADH suppresses triacylglycerol peroxidation in vitro. Notably, inducing polyunsaturated fatty acid-rich LDs triggers triacylglycerol peroxidation and LD-initiated ferroptosis when FSP1 activity is impaired. These findings uncover the first LD lipid quality-control pathway, wherein LD-localized FSP1 maintains neutral lipid integrity to prevent the build-up of oxidized lipids and induction of ferroptosis.

## Abstract (web summary)

Lange et al. identify a lipid droplet quality control pathway in which FSP1 safeguards stored neutral lipids from lipid peroxidation, thereby preventing the induction of ferroptosis.

## Main

Lipid droplets (LDs) are the primary cellular organelle for lipid storage^1,2^. During LD biogenesis, neutral lipids such as triacylglycerols (TGs) and cholesteryl esters (CEs) accumulate in the endoplasmic reticulum (ER) bilayer, forming a lipid lens that eventually buds into the cytoplasm^1,3^. LDs contain a neutral lipid core encircled by a phospholipid monolayer decorated with regulatory proteins^1,4^. Functionally, LDs serve as dynamic lipid storage depots, supplying lipids for biosynthesis and energy while protecting cells by sequestering potentially harmful species^1,5,6^.

Cells employ sophisticated quality-control mechanisms to preserve the integrity of biomolecules, including ubiquitin-dependent protein degradation^7^ and DNA damage repair^8^. Lipids are also vulnerable to damage, such as oxidation, necessitating lipid quality-control systems to prevent and counteract such damage^9^. Characterized by unchecked phospholipid peroxidation and consequent plasma membrane rupture, ferroptosis is an iron-dependent form of regulated cell death that represents a failure of lipid quality control^9–14^. To suppress ferroptosis, cells rely on specialized lipid quality-control mechanisms. The glutathione-dependent peroxidase GPX4 plays a pivotal role by converting lipid peroxides into benign lipid alcohols^15,16^. In parallel, FSP1 suppresses ferroptosis by recycling quinone antioxidants (CoQ10^17,18^ and vitamin K^19,20^), which quench lipid radicals and block their propagation.

Cells also modulate ferroptosis sensitivity by regulating membrane lipid composition. Polyunsaturated fatty acids (PUFAs) are prone to peroxidation compared to monounsaturated fatty acids (MUFAs), and increasing the phospholipid MUFA:PUFA ratio reduces ferroptosis sensitivity across various contexts^21,22^. This shift can be achieved by supplementing cells with excess MUFAs (for example, oleate)^23^ or by the regulation of lipid metabolic enzymes such as acyl-CoA synthetase (ACSL)-dependent fatty acid activation^24–26^ or membrane-bound *O*-acyltransferase (MBOAT) family-dependent fatty acid incorporation into phospholipids^27–29^. LDs have also been implicated in the regulation of phospholipid MUFA:PUFA composition. By sequestering PUFAs in stored TGs, LDs restrict their incorporation into membrane phospholipids, thereby decreasing membrane oxidizability and reducing cellular susceptibility to ferroptosis^10–12,14^.

The mechanisms underlying membrane phospholipid peroxidation have been extensively studied, but much less is known about the susceptibility of LD-residing neutral lipids to peroxidation. Whether neutral lipids within LDs are prone to oxidative damage and what cellular mechanisms exist to protect them remain largely unexplored. In this article we uncover a lipid quality control pathway for LDs in which FSP1 acts at LDs to safeguard stored neutral lipids from peroxidation and prevent LD-initiated ferroptosis.

## Results

### FSP1 is required to maintain PUFA-containing di- and triacylglycerols

**Fig. 1:**
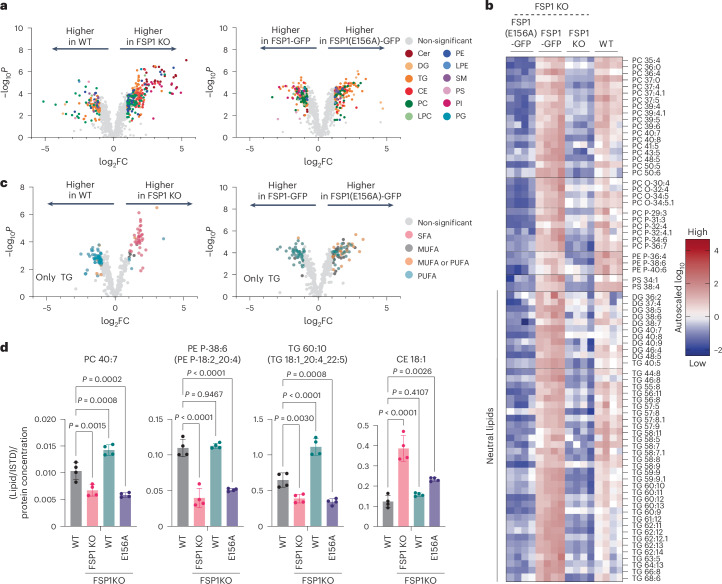
Loss of FSP1 results in lipid remodelling and a reduction in PUFA-containing glycerolipids. **a**, Volcano plots of untargeted lipidomics data comparing the global lipidomes of WT versus FSP1 KO and FSP1-GFP versus FSP1(E156A)-GFP re-expression constructs (GFP, green fluorescent protein). Lipid classes are coloured according to the legend. Cer, ceramide; DG, diacylglcyerol; TG, triacylglycerol; CE, cholesteryl ester; PC, phosphatidylcholine; LPC, lysophosphatidylcholine; PE, phosphatidylethanolamine; LPE, lysophosphatidylethanolamine; SM, sphingomyelin; PS, phosphatidylserine; PI, phosphatidylinositol; PG, phosphatidylglycerol. Data are autoscaled and log_10_-transformed. Thresholds for significant regulation are fold change (FC) > 2. Statistical significance was assessed by two-sided *t*-test, and values were deemed significant if *P* < 0.05 (false discovery rate (FDR)-corrected)). **b**, Heatmap displaying a cluster of lipids that are protected by catalytically active FSP1 as identified by hierarchical clustering in Extended Data Fig. 1d. Data are autoscaled and log_10_-transformed. **c**, Volcano plots of untargeted lipidomics data comparing TG lipids of WT versus FSP1 KO and FSP1-GFP versus FSP1(E156A)-GFP re-expression constructs. TG unsaturation is coloured according to the legend. SFA, saturated fatty acid containing (total double bonds = 0); MUFA, monounsaturated fatty acid containing (total double bonds = 1); PUFA, polyunsaturated fatty acid containing (total double bonds ≥ 4); MUFA or PUFA containing (total double bonds = 2 or 3). Data are autoscaled and log_10_-transformed. Thresholds for significant regulation are fold change > 2. Statistical significance was assessed by two-sided *t*-test, and values were deemed significant if *P* < 0.05 (FDR-corrected)). **d**, Selected lipids from the FSP1-protected lipid cluster. ISTD, internal standard. Raw data are plotted. Data are presented as mean values ± s.d., *n* = 4. Statistical significance is assessed by one-way analysis of variance (ANOVA; *P < 0.05). Source data

**Extended Data Fig. 1:**
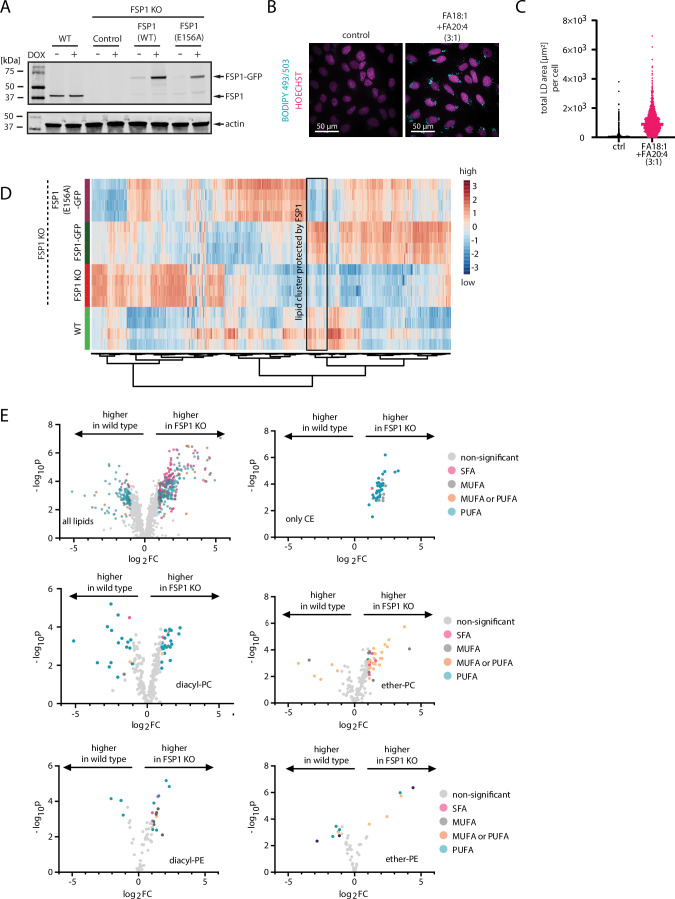
Loss of FSP1 result in lipid remodeling and a reduction in PUFA-containing glycerolipids. **A**: Western blot to assess FSP1 and FSP1-GFP expression levels in the indicated cell lines. DOX = doxycycline). **B** Lipid droplet imaging of wild type cells treated with oleate (FA 18:1) and arachidonate (FA 20:4). Lipid droplets were stained with BODIPY 493/503 and nuclei with Hoechst. **C**: Lipid droplets were imaged and lipid droplet area for each cell was quantified using Harmony data analysis software (Revvity). Each dot represents the lipid droplet area in an individual cell. **D**: Heatmap displaying all lipids quantified in each cell line. Hierarchical clustering was performed using Metaboanalyst 5.0 and a cluster of lipids specifically protected by FSP1 is indicated by the black box. (data was autoscaled and log10 transformed). **E**: Volcano plots of untargeted lipidomics data comparing all lipids, only CEs, only diacyl-phosphatidylcholine, only ether-phosphatidylcholine (O and P linked), diacyl-phosphatidylethanolamines, or ether-phosphatidylethanolamines (O and P linked) of wild type vs FSP1 KO cells. TG unsaturation is coloured according to legend. SFA = saturated fatty acid containing (total double bonds = 0), MUFA = monounsaturated fatty acid containing (total double bonds = 1), PUFA = polyunsaturated fatty acid containing (total double bonds ≥ 4), MUFA or PUFA (total double bonds = 2 or 3). (data was autoscaled and log10 transformed, thresholds for significant regulation are fold change > 2, statistical significance was assessed by two-sided t-test, values were deemed significant if p < 0.05 (FDR corrected)). Source data

To advance our understanding of the specific lipids protected by FSP1, we performed untargeted lipidomics to analyse the lipid profiles of FSP1 knockout (KO) cells and KO cells expressing doxycycline (DOX)-inducible wild-type (WT) or catalytically inactive E156A mutant FSP1 (Fig. 1 and Extended Data Fig. 1a). FSP1 is a key ferroptosis resistance factor in U-2 OS osteosarcoma cells^18^. However, the basal levels of neutral lipids and LDs in this cell type are low, limiting the ability to assess the potential role of FSP1 in the regulation and peroxidation of stored neutral lipids. Therefore, to increase neutral lipids and LDs, we supplemented the cells with a mixture of oleate (fatty acid (FA) 18:1) and arachidonate (FA 20:4) (3:1 ratio) (Extended Data Fig. 1b,c).

Lipidomics revealed extensive alterations in lipid abundance across the experimental conditions (Fig. 1a and Extended Data Fig. 1d). Unbiased hierarchical clustering identified a subset of lipids that were preserved, or protected, by active FSP1 but not the catalytically inactive E156A mutant FSP1 (Fig. 1b and Extended Data Fig. 1d; representative examples are shown in Fig. 1d). This cluster of lipids included highly unsaturated phospholipids, such as phosphatidylcholine (PC), phosphatidylethanolamine (PE) and phosphatidylserine (PS), as well as many neutral lipids, consistent with dual membrane and LD protective roles (Fig. 1b). FSP1 KO cells showed a shift from PUFA-TGs to saturated TGs (Fig. 1a–c and Extended Data Fig. 1e), revealing its role in maintaining unsaturated neutral lipids.

In addition to the lipids that decreased upon FSP1 KO, a subset of lipids showed increased levels. CEs were elevated in FSP1 KO cells, a change that was reversed by the expression of WT FSP1 and partially reversed by the catalytically inactive E156A mutant FSP1 (Fig. 1a,d and Extended Data Fig. 1e). CEs have been implicated in preventing TG lipid peroxidation as sacrificial antioxidants in vitro^30^, similar to the cholesterol intermediate 7-dehydrocholesterol^31–33^, suggesting that their increase may reflect an adaptive compensatory mechanism. We also observed an increase in some MUFA-TG species, particularly in cells expressing E156A mutant FSP1. The importance of this change is unclear, but would be predicted to be protective and could similarly indicate an adaptive mechanism.

### FSP1 is required to prevent oxidative damage to cellular PUFA-containing neutral lipids

**Fig. 2:**
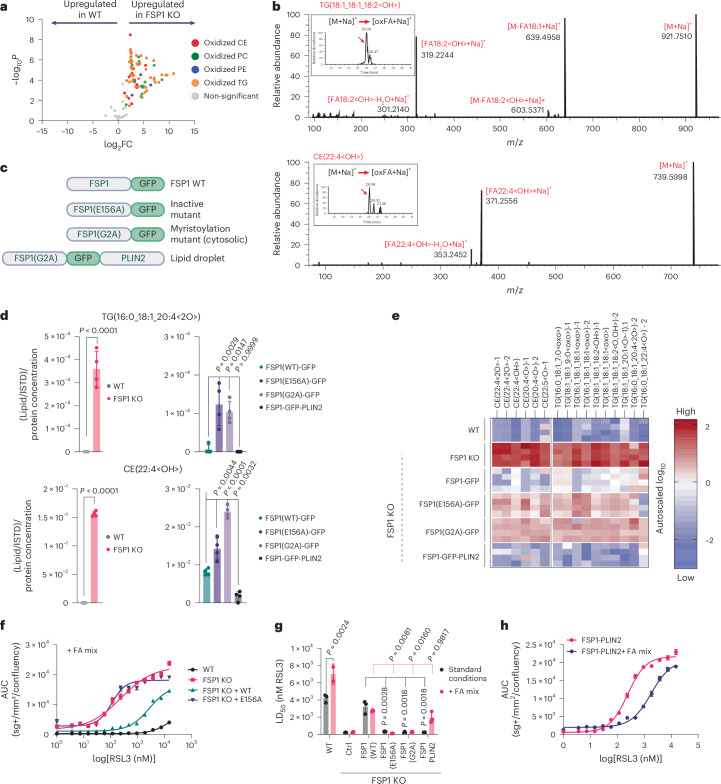
LD-localized FSP1 prevents neutral lipid peroxidation and ferroptosis. **a**, Volcano plots of untargeted epilipidomics data comparing oxidized lipids of WT versus FSP1 KO. Oxidized lipid class is coloured according to the legend. Data are autoscaled and log_10_-transformed. Thresholds for significant regulation are fold change > 2. Statistical significance was assessed by a two-sided *t*-test, and values were deemed significant if *P* < 0.05 (FDR-corrected). **b**, Example MS/MS spectra from parallel reaction monitoring-based analysis of sodiated ions of oxidized TG and CE displaying informative fragments necessary for oxidized TG and CE identification. Inset: parallel reaction monitoring-based quantification of the targeted transition (applied mass accuracy for extracted ion chromatogram generation = 5 ppm). **c**, Cartoon depiction of constructs re-expressed in FSP1 KO cells to study the role of FSP1 catalytic activity and subcellular localization. **d**, Selected oxidized lipids significantly elevated by FSP1 KO. Raw data are plotted, and data are presented as mean ± s.d., *n* = 4. Statistical significance was assessed by one-way ANOVA (*P < 0.05; NS, not significant). **e**, Heatmap displaying only oxidized TG and oxidized CE lipids extracted from a cluster of oxidized lipids that are protected by catalytically active FSP1, as identified by hierarchical clustering in Extended Data Fig. 3a. Data are autoscaled and log_10_-transformed. **f**, Cell death sensitivity to RSL3, assessed by time-resolved fluorescence microscopy (IncuCyte) with the cell death indicator SYTOX Green over the course of 24 h. The number of SYTOX Green-positive objects per image equals the number of dead cells and is normalized to cell area per image, expressed as confluency. sg+ indicates SYTOX Green-positive objects. Data are represented as mean ± s.e.m., *n* = 3. **g**, LD_50_ (lethal dose, 50%) values for RSL3 in different cell lines under standard conditions or LD-inducing conditions, determined by time-resolved fluorescence microscopy (IncuCyte) using SYTOX Green. LD_50_ values are calculated as the inflection point from the RSL3 dose–response curve under each condition. FA18:1, oleate; FA20:4, arachidonate. Data are presented as mean ± s.d., *n* = 3. Statistical significance was assessed by one-way ANOVA. *P < 0.05; NS, not significant. **h**, Cell death sensitivity to RSL3 assessed by time-resolved fluorescence microscopy (IncuCyte) with SYTOX Green over the course of 24 h. The number of SYTOX Green-positive objects per image equals the number of dead cells and is normalized to cell area per image expressed as confluency. sg+, SYTOX Green-positive objects. Data are presented as mean ± s.e.m., *n* = 3). Source data

A potential explanation for the observed reduction in PUFA-containing TGs in FSP1 KO cells (Fig. 1) is that they are being oxidized and thus no longer detected. Given the immense complexity of lipid species and their oxidized derivatives, we employed a semi-targeted liquid chromatography–tandem mass spectrometry (LC–MS/MS) approach for sample-specific epilipidome profiling^19,31,34,35^ for the measurement of oxidized lipids enriched in FSP1 KO cells. We detected numerous oxidized neutral lipids that selectively accumulated in FSP1 KO cells, including oxidized TGs and CEs (Fig. 2a,b). These findings implicate FSP1 as a key factor in protecting neutral lipids from oxidative damage.

**Extended Data Fig. 2:**
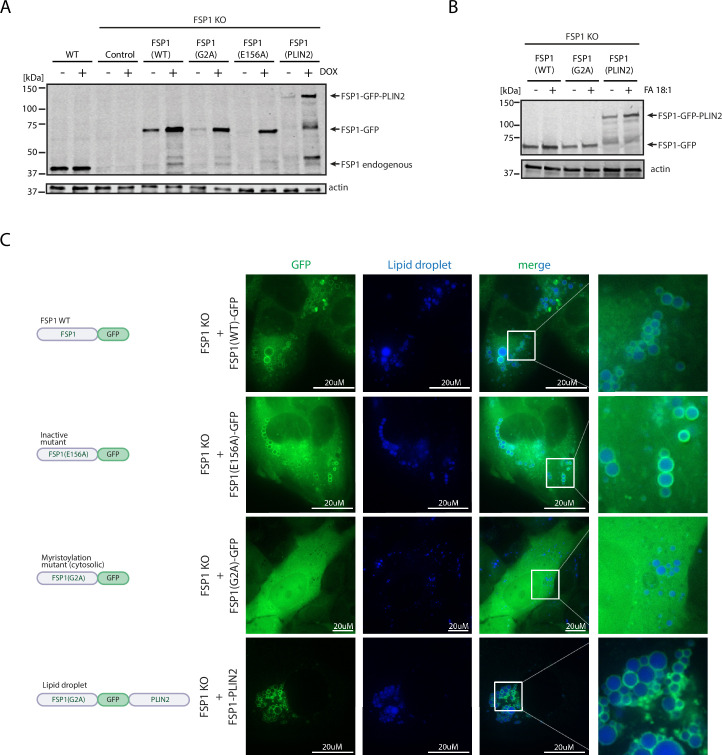
Lipid droplet-localized FSP1 prevents neutral lipid peroxidation. **A**: Western blot to assess FSP1 expression levels in used cell lines. DOX = doxycycline. Data shown are representative of three independent biological replicates. **B**: Western blot to assess FSP1 expression levels in cells in the presence and absence of mixed fatty acid supplementation (all cells grown in the presence of 10 ng/ml doxycycline for 48 h). Data shown are representative of three independent biological replicates. **C**: Epifluorescence microscopy to verify subcellular localization of FSP1 in utilized mutant cell lines. Cells were treated with 10 ng/ml doxycycline for 24 h followed by 100 µM FA18:1 for 24 h. Lipid droplets were stained with LipiBlue and FSP1-GFP constructs were visualized using green fluorescence. Data shown are representative of two independent biological replicates. Source data

**Extended Data Fig. 3:**
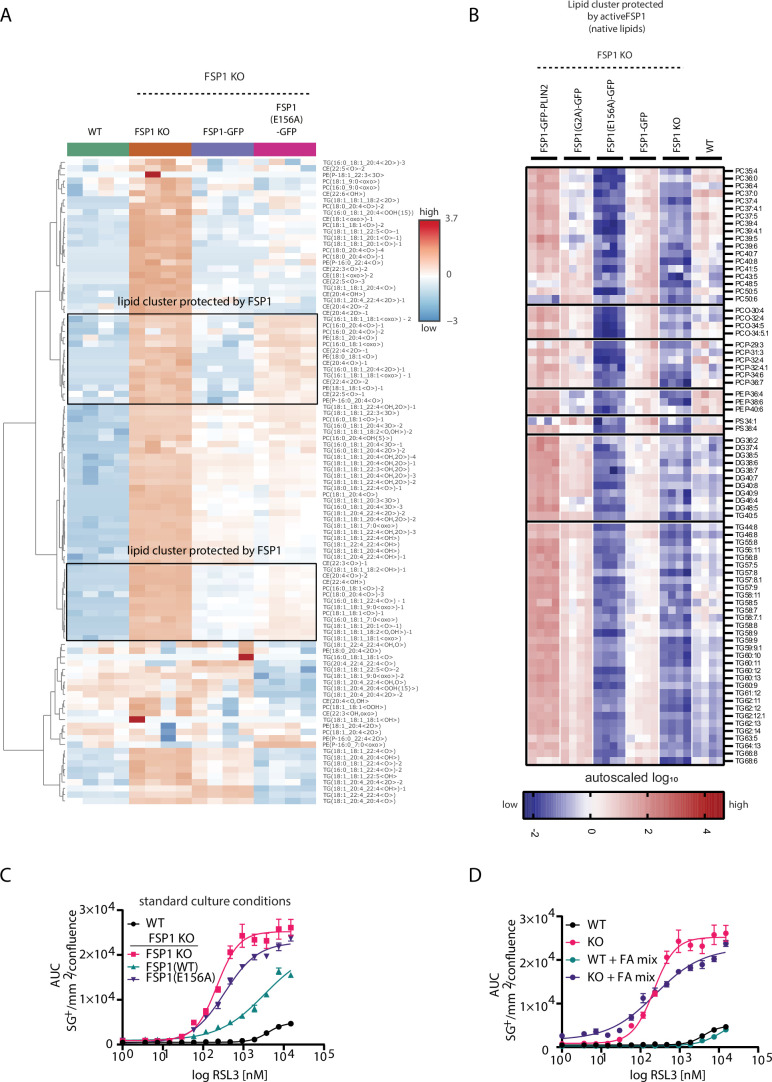
Lipid droplet-localized FSP1 prevents ferroptosis. **A**: Abundances of all oxidized lipids identified and quantified within different FSP1 expression constructs. Hierarchical clustering was performed using Metaboanalyst 5.0 and a cluster of lipids specifically protected by FSP1 is indicated by the black box (data was autoscaled and log10 transformed). **B**: Heatmap displaying lipid levels within different localization mutants from a cluster of lipids that is protected by catalytically active FSP1 as identified by hierarchical clustering in Extended Data Fig. 1E (data was autoscaled and log10 transformed). **C**, **D**: Cell death sensitivity to RSL3 assessed by time resolved fluorescence microscopy (incucyte) with cell death indicator sytox green over the course of 24 h. Number of sytox green positive objects per image equals the number of dead cells and is normalized to cell area per image expressed as confluency. sg + = sytox green positive objects. (data are represented as mean ± SEM, n = 3). Source data

FSP1 localizes to multiple organelles, including LDs^17,18^, raising the possibility that FSP1 influences oxidative damage to distinct sets of lipids at these discrete subcellular sites. To investigate the possibility that FSP1 acts directly on LDs, we analysed cell lines expressing a panel of FSP1 constructs^18^ (Fig. 2c and Extended Data Fig. 2a,b), including WT FSP1, catalytically inactive E156A mutant FSP1, myristoylation-defective G2A mutant FSP1, which impairs FSP1 membrane and LD recruitment, and a construct targeting FSP1 selectively to LDs (that is, FSP1 fused to PLIN2). Expression of WT FSP1 substantially suppressed the loss of PUFA-containing glycerolipids and the peroxidation of TGs and CEs in FSP1 KO cells (Fig. 2d,e and Extended Data Fig. 3a,b). In contrast, the E156A and G2A mutants exhibited diminished protective effects, highlighting the importance of FSP1’s enzymatic activity and membrane association (Fig. 2d,e). Remarkably, FSP1 targeted selectively to LDs by fusion to PLIN2 strongly suppressed TG and CE peroxidation (Fig. 2d.e), suggesting that FSP1 acts directly at LDs to preserve the integrity of stored TGs and CEs.

FSP1 is well established as a ferroptosis suppressor^17,18^, but its specific roles in LD function and the cellular consequences of oxidized neutral lipids are unknown. WT FSP1, but not the catalytically inactive E156A mutant, conferred resistance to RSL3-induced ferroptosis in FSP1 KO cells grown under both standard conditions (Extended Data Fig. 3c,d) or under fatty acid-supplemented conditions that induce LD biogenesis (Fig. 2f). Our previously published results indicated that plasma membrane-targeted FSP1 was sufficient to suppress ferroptosis, but that LD-targeted FSP1 had no effect of ferroptosis resistance^18^. However, those assays were performed under standard growth conditions^18^, in which U-2 OS cells have low TG levels and few LDs. To investigate the impact of LD-localized FSP1 and neutral lipid peroxidation on ferroptosis, we performed dose–response analyses for RSL3 in our panel of FSP1-targeted cell lines pretreated with fatty acids to induce LDs. As anticipated, WT FSP1 prevented RSL3-induced cell death under both standard and fatty acid-supplemented conditions, whereas the E156A and G2A mutants failed to confer protection (Fig. 2g and Extended Data Fig. 3c,d). Interestingly, although LD-targeted FSP1 (that is, FSP1-PLIN2) exhibited no protective effect under standard growth conditions, consistent with our previous results^18^, it suppressed ferroptosis under fatty acid-supplemented conditions in which cells contain LDs (Fig. 2g,h). These findings highlight the context-specific role of LD-localized FSP1 in protecting stored neutral lipids from oxidative damage and suppressing ferroptosis.

### CoQ10 is present in LDs isolated from cells

**Fig. 3:**
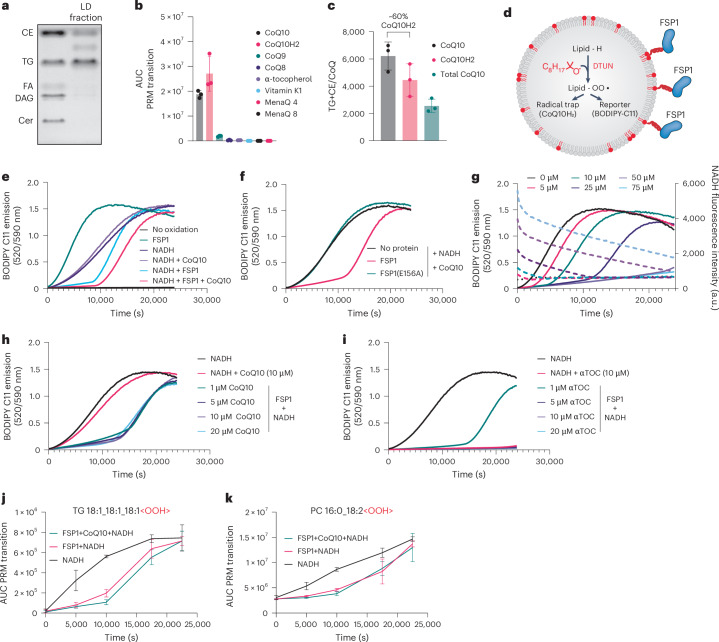
FSP1 reduction of CoQ10 suppresses TG peroxidation in artificial LDs. **a**, Thin-layer chromatogram of lipids extracted from LD fractions. **b**, Parallel reaction monitoring (PRM) used for the relative quantification of known FSP1 substrates in LD fractions generated by density gradient centrifugation from cell lysates. Data are presented as mean ± s.d., *n* = 3. **c**, Absolute quantification of CoQ10 levels by PRM in LD fractions spiked with an isotopically labelled CoQ10 internal standard. Levels of CoQ10 were normalized to total amounts of TG and CE lipids, quantified via thin-layer chromatography. Data are presented as mean ± s.d., *n* = 3. **d**, Cartoon describing the FENIX assay, adapted for artificial LDs after recruitment of recombinant FSP1. **e**, FENIX assay in artificial LDs with various substrate and enzyme compositions. **f**, FENIX assay in artificial LDs in the presence and absence of catalytically active FSP1 or the catalytically inactive FSP1(E156A) mutant. **g**, FENIX assay in artificial LDs in the presence of 100 nM FSP1, 10 µM CoQ10, 1 mM total lipid and varying concentrations of NADH. NADH consumption throughout the FENIX assay was assessed via NADH autofluorescence within the same experiment. **h**,**i**, FENIX assay in artificial LDs in the presence of increasing concentrations of CoQ10 (**h**) or α-tocopherol (**i**, αTOC). **j**,**k**, PRM-based quantification of TG and PC oxidation products during the peroxidation of artificial LDs. Data are presented as mean ± s.d., *n* = 3. Source data

To investigate the presence of FSP1 substrates in intracellular LDs, we established a targeted LC–MS/MS method to detect derivatives of coenzyme Q, vitamin K and vitamin E in LD-enriched buoyant fractions. As anticipated, these fractions were highly enriched with TG and CE (Fig. 3a). Among the quinone antioxidants, only CoQ10 (and small amounts of CoQ9) was detected in the LD fractions (Fig. 3b). To gain quantitative insights, we developed an absolute quantification method by spiking in isotopically labelled CoQ10, enabling the precise measurement of oxidized CoQ10, reduced CoQ10H2 and total CoQ10 levels in LD-enriched fractions. LD CoQ10 was present at ~1 per 2,000 neutral lipid molecules, with ~60% of CoQ10 in the reduced form (CoQ10H2) (Fig. 3c). These findings raise the possibility that FSP1 acts through local recycling of CoQ10 to maintain LD antioxidant capacity and prevent neutral lipid peroxidation.

### FSP1 suppresses oxidative damage to TG in a biochemically reconstituted system

**Extended Data Fig. 4:**
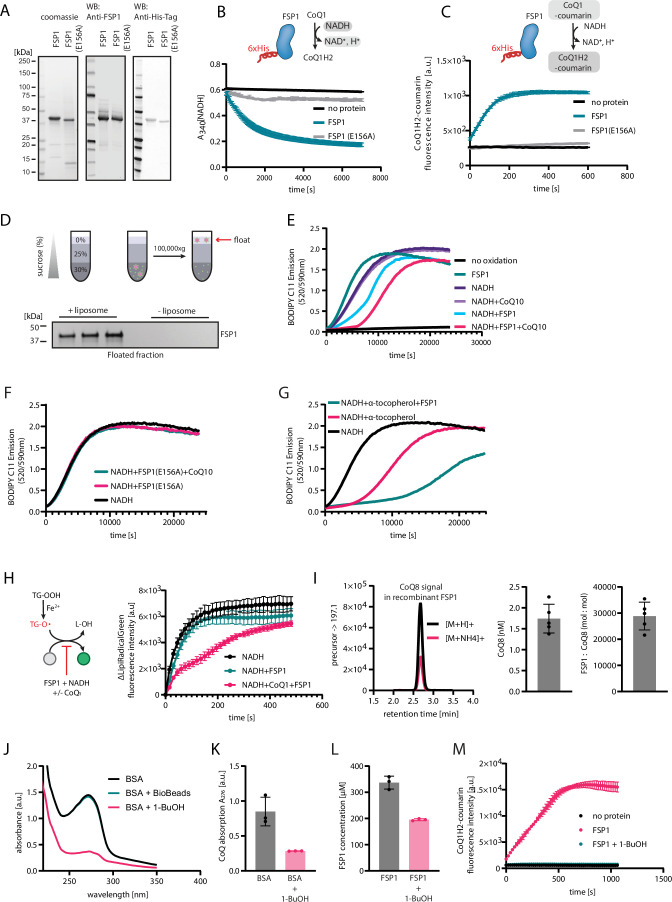
Recombinant FSP1 reduces CoQ10 by consuming NADH to suppress phospholipid peroxidation in liposomes in vitro. **A**: Characterization of recombinantly expressed FSP1. Coomassie stained gel indicates high FSP1/FSP1(E156A) purity and western blot analysis proves the identity of FSP1 and the presence of a His-Tag. **B**: Recombinant FSP1 but not FSP1(E156A) is capable of consuming NADH in the presence of the soluble CoQ10 derivative CoQ1. NADH consumption is assessed by measuring endogenous absorbance of NADH at 340 nm. (data are represented as mean ± SD, n = 3) **C**: Recombinant FSP1 but not FSP1(E156A) can reduce CoQ to CoQH2 in the presence of NADH. CoQH2 formation is assessed using the fluorescent CoQ-reduction reporter CoQ1-coumarin. (data are represented as mean ± SD, n = 3) **D**: Recombinant FSP1 can be recruited to nickel-phospholipid doped liposomes. Recruitment was assessed using a liposome flotation assay and gel electrophoresis with silver stain detection. Data shown are representative of two independent replicates. **E**: FENIX assay is Egg-phosphatidylcholine liposomes in the presence of various substrate and enzyme combinations. (data are represented as mean, n = 3) **F**: FENIX assay in Egg-phosphatidylcholine liposomes in the presence of catalytically inactive FSP1(E156A). (data are represented as mean, n = 3) **G**: FENIX assay in Egg-phosphatidylcholine liposomes in the presence of α-tocopherol and/or FSP1. (data are represented as mean, n = 3) **H**: LipiRadicalGreen assay to detect free radicals generated by the reaction of triglyceride hydroperoxides with iron. LipiRadicalGreen activation was monitored using fluorescence spectroscopy. (data are represented as mean ± SD, n = 3) **I**: LC-MS/MS chromatogram of the transition of CoQ8 [M + H]+ and [M + NH4]+ adducts to m/z 197.1 for and quantification of CoQ8 and CoQ10 levels in recombinant FSP1 preparations. (data are represented as mean ± SD, n = 3) **J**: Detection of BSA bound CoQ10 as a model system for copurified coenzyme Q in recombinant FSP1 preparations. CoQ10 was detected via absorbance spectroscopy. BSA/CoQ10 complex was treated with biobeads or 1-butanol (1-BuOH) to delipidate BSA. **K**: BSA/CoQ10 complex delipidated with 1-butanol. Remaining CoQ10 levels were determined by absorbance at 260 nm. (data are represented as mean ± SD, n = 3) **L**: Recombinant FSP1 was treated with 1-butanol and protein levels remaining after delipidation were quantified via bichinonic acid assay. (data are represented as mean ± SD, n = 3) **M**: Measurement of remaining FSP1 activity after delipidation via CoQ1-coumarin assay. (data are represented as mean ± SD, n = 3). Source data

To examine the mechanisms of FSP1 in suppressing lipid peroxidation, we used biochemically reconstituted approaches. Purified recombinant His-tagged WT FSP1, but not the E156A mutant, displayed the expected activity, consuming NADH in the presence of the soluble CoQ10 analogue CoQ1 and reducing the soluble and fluorescent CoQ10 analogue CoQ1-coumarin to its quinol form in the presence of NADH (Extended Data Fig. 4a–c). To determine whether recombinant FSP1 can suppress lipid peroxidation using CoQ10, we generated liposomes containing nickel-conjugated phospholipids to recruit His-tagged FSP1 to the liposome surface (Extended Data Fig. 4d). Employing the FENIX (fluorescence-enabled inhibited autoxidation) assay^36^, lipid peroxidation was triggered using the lipid radical initiator di-*tert*-undecyl hyponitrite (DTUN) and lipid peroxidation propagation followed by BODIPY-C11 fluorescence (Extended Data Fig. 4e). In this reconstituted liposome system, WT FSP1, but not the E156A mutant, prevented lipid peroxidation when its substrates NADH and CoQ10 were present (Extended Data Fig. 4e,f). FSP1 also efficiently suppressed lipid peroxidation in the presence of NADH and the vitamin E derivative α-tocopherol (Extended Data Fig. 4g).

When carefully examining the controls, we noted that FSP1 rescued lipid peroxidation in liposomes and artificial LDs in the absence of exogenously added CoQ10. We wondered whether FSP1 directly reduces lipid radicals, and we examined this possibility by tracking the formation of TG alkoxyl radicals using LipiRadicalGreen^37^. However, FSP1 only reduced lipid radicals in the presence of CoQ and NADH, but not in the presence of NADH itself (Extended Data Fig. 4h), indicating that other mechanisms govern the observed background activity. We hypothesized that the recombinant FSP1 preparations contain low levels of CoQ that were copurified from bacteria. To test this hypothesis, we implemented a targeted quantification method for CoQ10 and its bacterial isoform CoQ8. We indeed identified CoQ8 in recombinant FSP1 preparations (Extended Data Fig. 4i). We tested two protocols to remove CoQ by using bovine serum albumin (BSA)/CoQ10 as a model system, and identified that protein-bound CoQ10 can be removed by the non-denaturing organic solvent 1-BuOH (Extended Data Fig. 4j). This workflow was applied to CoQ10/BSA and recombinant FSP1, and resulted in ~50% loss of protein concentration (Extended Data Fig. 4k,l) and a complete loss of FSP1 activity (Extended Data Fig. 4m), indicating that this protocol was incapable of effectively removing CoQ and simultaneously maintaining enzymatic function. Thus, although FSP1 suppresses phospholipid peroxidation, there is some background activity due to copurified bacterial CoQ8.

**Extended Data Fig. 5:**
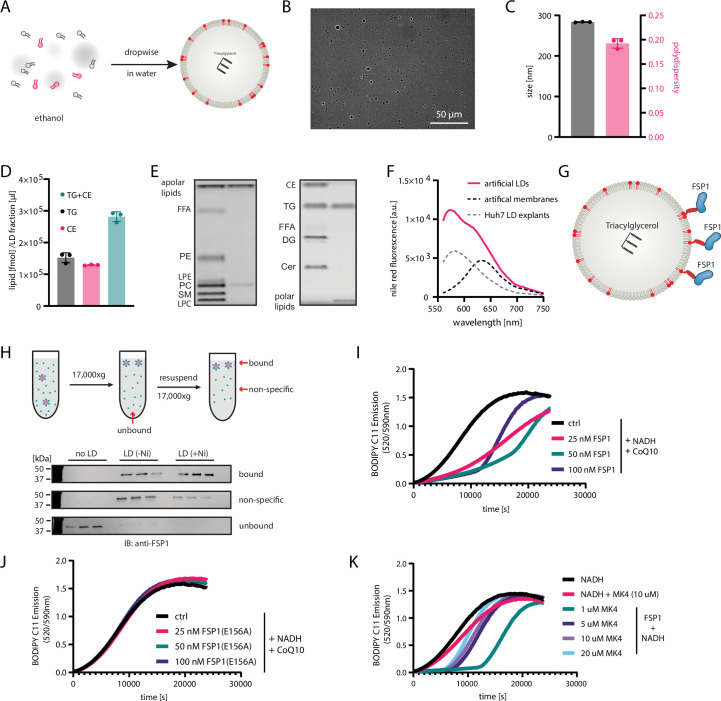
Artificial lipid droplets recapitulate cellular lipid droplets and recruitment of recombinant FSP1 prevents lipid peroxidation in vitro. **A**: Schematic of ethanolic infusion of lipids to synthesize artificial lipid droplets. **B**: Phase contrast microscopy of artificial lipid droplet preparations. **C**: Size and polydispersity of artificial lipid droplet preparations assessed by dynamic light scattering. (data are represented as mean ± SD, n = 3) **D**: Lipid class distribution in lipid droplet fractions extracted from U-2 OS cells treated with 200 µM oleate and 0.1% BSA for 24 h. Lipids were quantified by thin layer chromatography. (data are represented as mean ± SD, n = 3) **E**: Thin layer chromatograms of lipids artificial lipid droplets. **F**: Nile red fluorescence in liposomes, artificial lipid droplets and lipid droplets extracted from Huh7 cells. Nile red was excited at 530/10 nm and shows red-shifted fluorescence in comparison to liposomes but similar fluorescence as compared to lipid droplet fractions from cells indicating a lipid droplet like supramolecular structure. **G**: Schematic describing the strategy to recruit His-tagged FSP1 to nickel-phospholipid spiked artificial lipid droplets. Red phospholipids = DGS-NTA(Ni), red tail on FSP1 = His-Tag. **H**: Schematic describing artificial lipid droplet flotation to probe successful recruitment of His-tagged FSP1. Lower panel shows silver stained gel detecting FSP1 protein in each collected fraction indicating strong binding. **I**: FENIX assay in artificial lipid droplets in the presence of 100 nM FSP1, 25 µM NADH and varying concentrations of the alternative FSP1 substrate menaquinone-4 (MK4). (data are represented as mean, n = 3) Data shown are representative of two independent replicates. **J**: FENIX assay in artificial lipid droplets in the presence of 10 µM CoQ10, 25 µM NADH and varying concentrations of the catalytically dead FSP1 mutant FSP1(E156A). (data are represented as mean, n = 3) **K**: FENIX assay in artificial lipid droplets in the presence of 10 µM CoQ10, 25 µM NADH and varying concentrations of active FSP1 mutant FSP1. (data are represented as mean, n = 3). Source data

To test the ability of FSP1 to directly protect TG from oxidative damage in LDs, we developed a modified FENIX assay that uses artificial LDs generated in vitro (Fig. 3d and Extended Data Fig. 5a). Artificial LDs composed of TG cores and a phospholipid monolayer, ~290 nm in diameter (Extended Data Fig. 5b,c), were validated by thin-layer chromatography (Extended Data Fig. 5d,e) and the fluorescence emission spectrum of the solvatochromatic dye Nile Red (Extended Data Fig. 5f). Similar to our liposome assays, His-tagged FSP1 was recruited to the LD surface using nickel-conjugated phospholipids (Extended Data Fig. 5h). Following the initiation of lipid peroxidation with DTUN, we observed unrestricted lipid peroxidation based on the oxidation of BODIPY-C11 (Fig. 3e). The addition of NADH marginally reduced the rate of lipid peroxidation (Fig. 3e). However, NADH did not fully prevent lipid peroxidation, as indicated by the absence of a lag phase. In contrast, the addition of FSP1, together with CoQ10 and NADH, resulted in full inhibition of lipid peroxidation propagation, evident by an extended lag phase (Fig. 3e and Extended Data Fig. 5i). It should be noted that the background lipid peroxidation-suppressing function of FSP1 is also observed in artificial LD oxidations (Fig. 3e), and is even more pronounced than in liposomes. As expected, the E156A mutant FSP1 had no effect on the kinetics of lipid peroxidation (Fig. 3f and Extended Data Fig. 5j). In the presence of FSP1, NADH and CoQ10, the lag phase continued for approximately 10,000 s and then unrestricted lipid peroxidation was observed (Fig. 3e,f). These findings suggested that a component within the reaction was being consumed. Indeed, the addition of increasing concentrations of NADH resulted in an extension in the lag phase followed by lipid peroxidation upon complete consumption of NADH (Fig. 3h). In contrast, addition of higher amounts of CoQ10 did not affect the reaction (Fig. 3h), indicating that 1 µM CoQ10 is sufficient to drive maximal suppression of lipid peroxidation in this assay and that the low levels of copurified CoQ8 are already sufficient to drive near-maximal lipid peroxidation suppression. FSP1 was also able to suppress lipid peroxidation in the presence of NADH together with the vitamin E derivative α-tocopherol or the vitamin K derivative menaquinone-4 (Fig. 3i and Extended Data Fig. 5k). Given that we were unable to detect α-tocopherol or menaquinone-4 in LDs purified from our cells, it is likely that FSP1 uses CoQ10 to prevent neutral lipid peroxidation in LDs. However, there may be cell types or metabolic conditions in which α-tocopherol or menaquinone-4 are present in LDs, and FSP1 could employ these other quinone antioxidants to maintain LD lipid quality.

**Extended Data Fig. 6:**
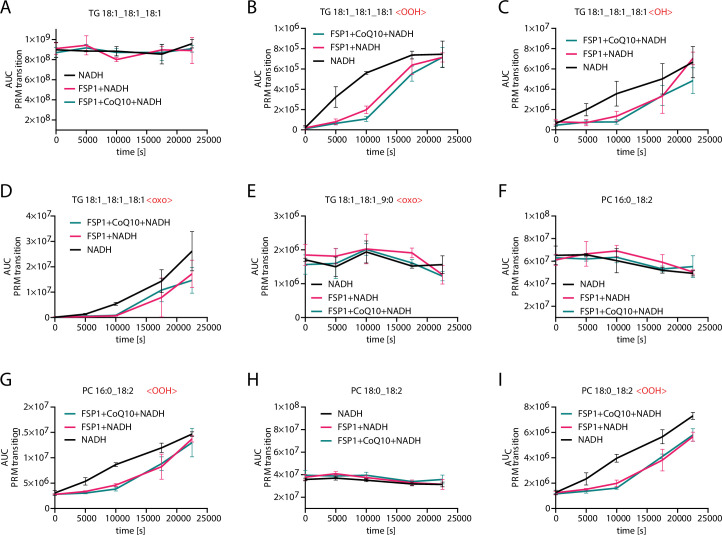
In vitro peroxidation of artificial lipid droplets is characterized by phospholipid and TG peroxidation and FSP1 prevents lipid peroxidation in a CoQ and NADH dependent manner. **A**–**I**: Quantification of lipid peroxidation products detected by parallel reaction monitoring during peroxidation of artificial lipid droplets in the presence of 25 µM NADH, 10 µM CoQ10 and 100 nM FSP1. (data are represented as mean ± SD, n = 3). Source data

In vitro-generated artificial LDs contain both phospholipids and TGs. An indirect lipid peroxidation indicator such as BODIPY-C11 is unable to discern which lipids are oxidized. Semi-targeted oxidized epilipidomics analyses revealed that FSP1, together with CoQ10 and NADH (but also NADH alone due to the background activity of copurified CoQ8), suppressed the oxidation of both TGs (Fig. 3j and Extended Data Fig. 6a–e) and phospholipids (Fig. 3k and Extended Data Fig. 6f–i). Thus, our findings in cellular and reconstituted systems indicate that FSP1 acts as a CoQ10 oxidoreductase to prevent oxidative damage to neutral lipids stored in LDs.

### PUFA supplementation generates PUFA-rich LDs that are prone to peroxidation

**Fig. 4:**
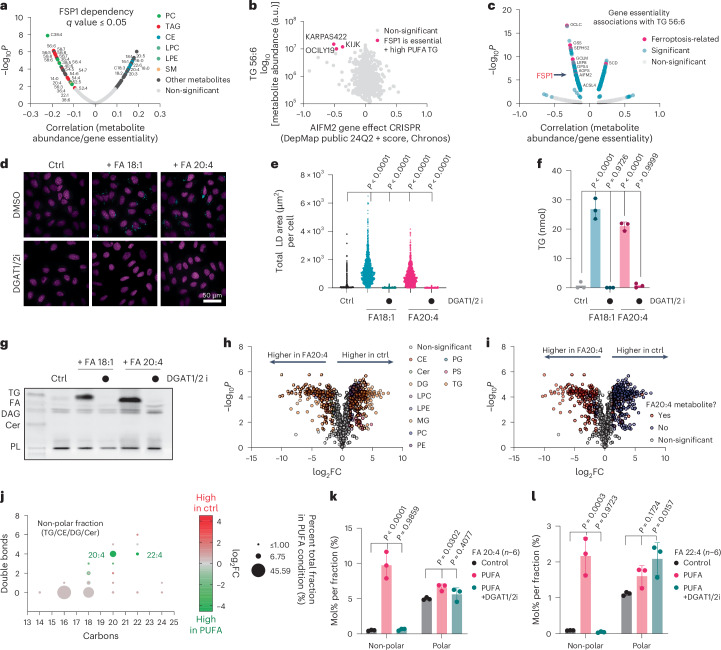
Exogenous arachidonate supplementation induces PUFA-rich LDs. **a**, Mining the cancer dependency portal DepMap for correlations of FSP1 essentiality with metabolite abundance indicates FSP1 essentiality correlates with elevated levels of PUFA-containing TG and PUFA-containing PC lipids. *q* values represent Benjamini–Hochberg FDR-adjusted *P* values for the association between metabolite abundance and gene essentiality (DepMap portal). **b**, Example correlation of TG 56:6 with FSP1 essentiality across 927 cancer cell lines. Each dot represents one cell line. **c**, Association of relative abundance of TG 56:6 with gene essentiality indicates that anti-ferroptotic genes correlate highly with PUFA-TG levels. *q* values represent Benjamini–Hochberg FDR-adjusted *P* values for the association between metabolite abundance and gene essentiality (DepMap portal). **d**, LD quantification in U-2 OS cells treated with 200 µM oleate (FA 18:1) or arachidonate (FA 20:4) for 24 h in the presence and absence of DGAT1 (15 μM A-922500) and DGAT2 (10 μM PF-06424439) inhibitors. LDs were stained with BODIPY 493/503 and nuclei with Hoechst. **e**, LDs were imaged, and the LD area for each cell was quantified using Harmony data analysis software (Revvity). Each dot represents the LD area in an individual cell. Statistical significance was assessed by one-way ANOVA. *P < 0.05; NS, not significant. **f**, Quantification of TG levels via thin-layer chromatography. Statistical significance was assessed by one-way ANOVA. *P < 0.05; NS, not significant. **g**, Example of a thin-layer chromatogram used for the quantification in **f**. **h**, LC–MS/MS-based untargeted lipidomics comparing cells in the presence and absence of 200 µM FA 20:4 for 24 h. Lipid classes are coloured according to the legend. Data were autoscaled and log_10_-transformed. Thresholds for significant regulation are fold change > 2. Statistical significance was assessed by a two-sided *t*-test, and values were deemed significant if *P* < 0.05 (FDR-corrected). **i**, The same data as displayed in **h**. Significantly regulated lipids are coloured based on the presence or absence of FA20:4 metabolites. A lipid was deemed a putative FA20:4 metabolite when either of its fatty acids had more than 20 carbons with more than four double bonds. Data are autoscaled and log_10_-transformed. Thresholds for significant regulation are fold change > 2. Statistical significance was assessed by a two-sided *t*-test, and values were deemed significant if *P* < 0.05 (FDR-corrected). **j**, Bubble plot showing absolute quantities and the regulation of fatty acids in the non-polar lipid fraction extracted from U-2 OS cells treated with 200 µM FA20:4 for 24 h. For quantification, lipid extracts were separated into a non-polar lipid fraction using solid-phase extraction, and esterified fatty acids were transmethylated and quantified using GC–FID. **k**, Mol% of arachidonate (FA20:4 (*n*–6)) in non-polar and polar lipid fractions. Data are presented as mean ± s.d., *n* = 3. Statistical significance was assessed by one-way ANOVA. *P < 0.05; NS, not significant. **l**, Mol% of adrenate (FA22:4 (*n*–6)) in non-polar and polar lipid fractions. Data are presented as mean ± s.d., *n* = 3. Statistical significance was assessed by one-way ANOVA. *P < 0.05; NS, not significant. Source data

Analysis of FSP1 dependency in DepMap revealed a correlation between FSP1 essentiality and the amount of PUFA-containing TGs across 927 cancer cell lines (Fig. 4a,b). Although, FSP1 is not essential in most cell types, FSP1 is required for the viability of a subset of cell types with high PUFA TGs (Fig. 4a,b). PUFA-containing TG levels (for example, TG 56:6) correlate with the essentiality for many genes related to ferroptosis, including genes involved in glutathione synthesis, selenoprotein biosynthesis, fatty-acid metabolism and antioxidant defence (for example, *GPX4* and *FSP1/AIFM2*) (Fig. 4c). These data indicate a correlation between PUFA-containing TGs and ferroptosis sensitivity, which may reflect the overall cellular lipid unsaturation environment or a vulnerability to ferroptosis conferred by the unsaturation of TGs.

**Extended Data Fig. 7:**
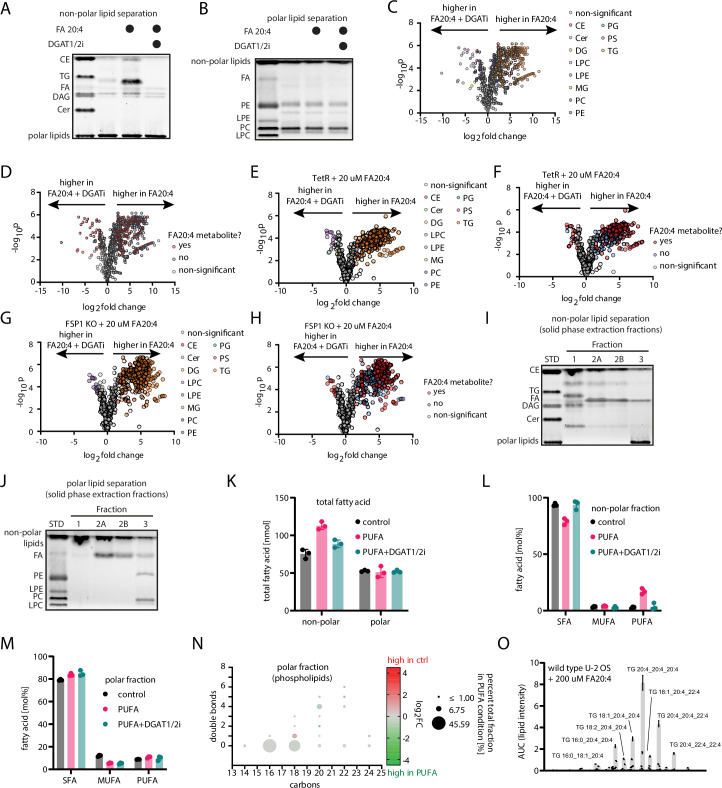
Arachidonic acid treatment induces specific accumulation of polyunsaturated non-polar lipids. **A**, **B**: Lipid extracts from cells treated with 200 µM FA20:4 for 24 h in the presence and absence of DGAT1/2 inhibitors (15 μM A-922500 and 10 μM PF-06424439). Non-polar and polar lipids are separated and quantified by thin layer chromatography. **C**, **E**, **G**: LC-MS/MS based untargeted lipidomics of conditions described in the graph titles. Lipid classes are colored according to legend. Cer = ceramide, DG = diacylglcyerol, TG = triacylglycerol, CE = cholesteryl ester, PC = phosphatidylcholine, LPC = lysophosphatidylcholine, PE = phosphatidylethanolamine, LPE = lysophosphatidylethanolamine, SM = sphingomyelin, PS = phosphatidylserine, PI = phosphatidylinositol, PG = phosphatidylglycerol. (data was autoscaled and log10 transformed, thresholds for significant regulation are fold change > 2, statistical significance was assessed by two-sided t-test, values were deemed significant if p < 0.05 (FDR corrected)). **D**, **F**, **H**: Same data as displayed in Extended Data Fig. 6A, C, E. Significantly regulated lipids are colored based on the presence or absence of FA20:4 metabolites. A lipid was deemed a putative FA20:4 metabolite when either of its fatty acids had more than 20 carbons with more than 4 double bonds. (data was autoscaled and log10 transformed, thresholds for significant regulation are fold change > 2, statistical significance was assessed by two-sided t-test, values were deemed significant if p < 0.05 (FDR corrected)) **I**, **J**: Lipid extracts from cells were separated into fractions and non-polar and polar lipids through solid phase extraction and subsequently separated by thin layer chromatography and imaged via primuline staining. Solid phase fractionation was carried out as follows – Fraction 1: non-polar lipids eluted with 4 ml chloroform/isopropanol (2:1, v/v), Fraction 2A: free fatty acids eluted with 4 ml diethylether/acetic acid (100:1, v/v), Fraction 2B: second free fatty acid fraction eluted with additional 4 ml diethylether/acetic acid (100:1, v/v), Fraction 3: polar lipids eluted with 4 ml methanol. **K**: Lipid extracts were separated, and esterified fatty acids were quantified. All fatty acid concentrations were summed and represent total fatty acid concentrations in each fraction. (data are represented as mean ± SD, n = 3) **L**, **M**: Concentrations of saturated fatty acids (SFA), monounsaturated fatty acids (MUFA) and polyunsaturated fatty acids (PUFA) in each fraction were summed up. (data are represented as mean ± SD, n = 3) **N**: Bubble plot showing absolute quantities and regulation of fatty acids in the polar lipid fraction extracted from U-2 OS cells treated with 200 µM FA20:4 for 24 h. For quantification, lipid extracts were separated into non-polar lipid fraction using solid phase extraction and esterified fatty acids were transmethylated and quantified using gas chromatography-flame ionization detection. **O**: Triacylglycerol lipid intensity from untargeted lipidomics indicating the simplicity of the triacylglycerol species under the studied conditions. Source data

**Extended Data Fig. 8:**
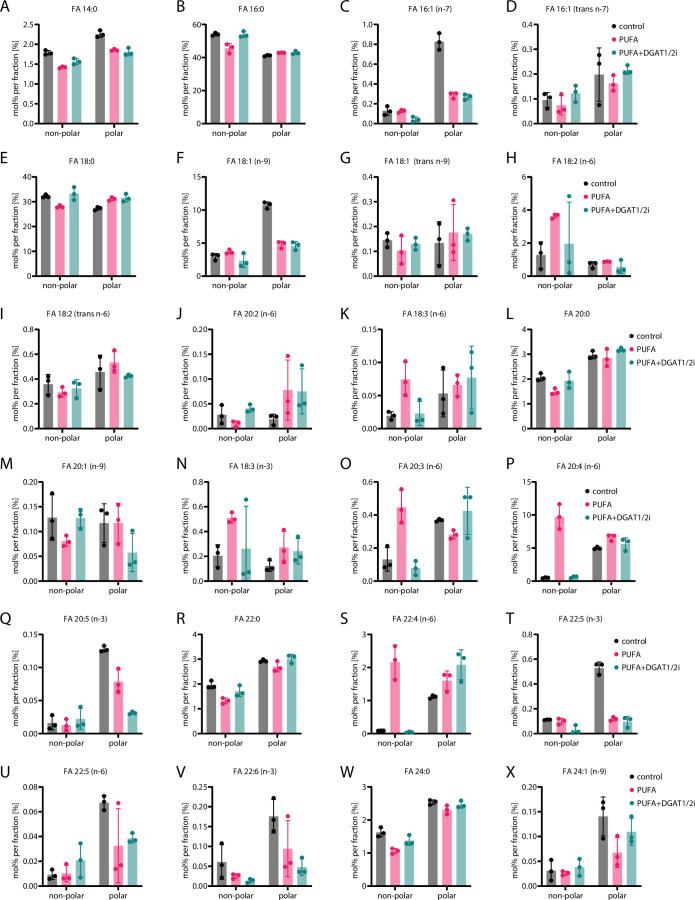
Arachidonic acid treatment induces specific accumulation of arachidonic acid containing non-polar lipids and is blocked by DGAT1/2 inhibition. **A**–**X**: Cells were treated with 200 µM FA20:4 for 24 h in the presence and absence of DGAT1/2 inhibitors (15 μM A-922500 and 10 μM PF-06424439). Lipids were extracted from cell pellets. Lipid extracts were separated into polar and non-polar lipids using solid phase extraction. Esterified fatty acids were released and transmethylated. Fatty acid methyl esters were absolutely quantified using gas chromatography coupled to flame ionization detection. (data are represented as mean ± SD, n = 3). Source data

To explore the possibility that PUFA-rich TG confers ferroptosis sensitivity and represents a vulnerability to the loss of FSP1, we developed a cell-based system in which we generate high amounts of PUFA-rich TGs. We hypothesized that FSP1 inactivation in this background would preferentially lead to LD peroxidation. Incubation of U-2 OS cells with the MUFA oleate (FA 18:1) or the PUFA arachidonate (FA 20:4) induced comparable biosynthesis of TGs and LDs, which were both dependent on diacylglycerol acyltransferase 1 and 2 (DGAT1/2) (Fig. 4d–f). Incubation with arachidonate altered the cellular lipid landscape, with the majority of changes being in the amounts of TG lipids (Fig. 4g,h and Extended Data Fig. 7). Analysis of TG lipid changes also revealed an overwhelming increase in TG lipids containing arachidonate or putative arachidonate elongation and desaturation products (Fig. 4i), which was dependent on DGAT1/2 (Extended Data Fig. 7). A small number of phospholipids were also upregulated under arachidonate-supplemented conditions, indicating that arachidonate was also incorporated into other membranes. The untargeted lipidomics approach utilized in this study does not provide quantitative information regarding fatty-acid quantities within different lipid classes, and only yields relative quantitative information for individual intact lipids. We thus extracted total lipids from cells, isolated non-polar lipids and polar lipids (Extended Data Fig. 7i,j), and used gas chromatography with flame ionization detection (GC–FID) to quantify the fatty-acid compositions (Fig. 4j–l and Extended Data Figs. 7k–n and 8). This analysis indicated an increase in arachidonate and adrenic acid (22:4)-containing non-polar lipids (Fig. 4j) in the treated samples relative to control, with very little change observed in the polar lipids (that is, phospholipids) (Extended Data Fig. 7n). Importantly, DGAT inhibition blocked the increase in arachidonate and adrenic acid in non-polar lipids, with little effect on the amount present in polar lipids (Fig. 4k,l). These data establish a cell-based system to generate intracellular LDs with high amounts of PUFA-containing TGs.

### Inhibition of FSP1 in cells containing PUFA-TG-rich LDs induces LD-specific neutral lipid peroxidation

**Fig. 5:**
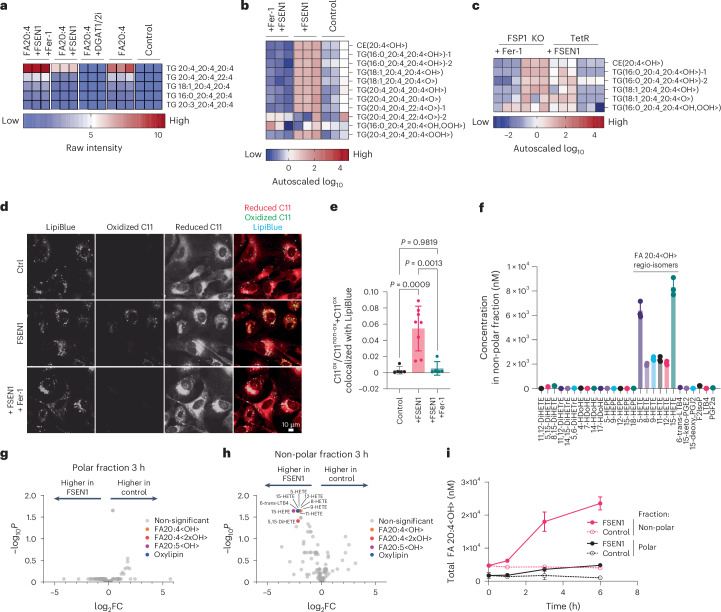
FSP1 prevents the peroxidation of PUFA-rich LDs. **a**, Highest intense TG lipids in cells treated with 200 µM FA20:4 for 24 h in the presence or absence of the indicated compounds, as quantified by untargeted lipidomics. **b**, Semi-targeted epilipidomics analysis of non-polar lipids in WT U-2 OS cells. The cells were cultured in medium supplemented with 200 µM FA20:4 for 24 h. After 24 h, the cells were treated with the indicated compounds for 6 h (shortly before cell death set in) and collected. **c**, Semi-targeted epilipidomics analysis of non-polar lipids in control and FSP1 KO U-2 OS cells treated with 20 µM FA20:4 for 24 h in the presence or absence of the indicated compounds. **d**, U-2 OS cells were cultured in medium supplemented with 200 µM FA20:4 for 24 h and subsequently treated with the indicated compounds. LDs were visualized using LipiBlue, and lipid peroxidation was visualized with BODIPY-C11. **e**, Quantification of the signal densities of colocalized markers for LDs (LipiBlue signal) and lipid peroxidation (BODIPY-C11) was performed using ImageJ. Data are presented as mean ± s.d., *n* = 7. Data were analysed using one-way ANOVA followed by Tukey’s post hoc test (two-sided), with adjustment for multiple comparisons. **f**, U-2 OS cells were cultured in medium supplemented with 200 µM FA20:4, followed by incubation with FSEN1, and the cells were collected at 6 h. Absolute quantification of oxidized fatty acids was performed by separating lipid extracts into polar and non-polar lipid fractions, followed by basic hydrolysis and targeted LC–MS/MS for absolute quantification of oxidized fatty acids. The oxidized fatty acids displayed were identified by hierarchical clustering (Extended Data Fig. 10a). HETE, hydroxy-eicosa-tetra-en-oic acid; DiHETE, dihydroxy-eicosa-tetra-en-oic acid; DiHETrE, dihydroxy-eicosa-tri-en-oic acid; HDoHE, hydroxy-docosa-hexa-en-oic acid; HEPE, hydroxy-eicosa-penta-en-oic acid; LTB, leukotriene B; PGE, prostaglandin E; IsoP, isoprostane. Data are presented as mean ± s.d., *n* = 3. **g**,**h**, U-2 OS cells were cultured in medium supplemented with 200 µM FA 20:4 for 24 h, followed by incubation with FSEN for 3 h. Oxidized fatty acids in polar lipids (**g**) or non-polar lipids (**h**) were quantified as described in **f**. Data are autoscaled. Thresholds for significant regulation are fold change > 2. Statistical significance was assessed by two-sided *t*-test, and values were deemed significant if *P* < 0.05 (FDR-corrected). **i**, U-2 OS cells were cultured in medium supplemented with 200 µM FA 20:4 for 24 h, followed by incubation with FSEN for 0, 1, 3 or 6 h. Oxidized fatty acids in polar or non-polar lipids were quantified as described in **f**. All HETE regioisomers were summed up and plotted over time. Data are presented as mean ± s.d., *n* = 3. Source data

We hypothesized that the PUFA-rich TGs in arachidonate-treated cells would be peroxidation-prone and sensitive to the loss of FSP1 function. Indeed, untargeted lipidomics indicated that treatment with the FSP1 inhibitor FSEN1 reduced the highest abundant arachidonate-containing TGs, implying their consumption by lipid peroxidation (Fig. 5a and Extended Data Fig. 7o). Co-treatment with the radical-trapping antioxidant ferrostatin (Fer-1) reversed this decrease (Fig. 5a), indicating that the observed decrease in arachidonate-containing TGs following FSP1 inhibition is peroxidation-dependent. To test this possibility directly, we performed semi-targeted epilipidomics analysis. Treatment with the FSP1 inhibitor FSEN1 resulted in the accumulation of oxidized arachidonate-containing CE and TG lipids, which was blocked by co-treatment with Fer-1, indicating that oxidized TG and CE are generated by active lipid peroxidation (Fig. 5b). To exclude possible off-target effects of FSP1 inhibitors, we also performed a similar assay in FSP1 KO cells. FSP1 KO cells display heightened cell death sensitivity to arachidonate treatment, so a lower arachidonate concentration had to be used to induce a PUFA TG phenotype to maintain the viability of the FSP1 KO cells. Treatment of the FSP1 KO cells with 20 μM arachidonate induced a similar upregulation of TGs containing arachidonate and putative arachidonate metabolites that could be blocked with DGAT1/2 inhibitors (Extended Data Fig. 7c–h). Within this system, FSP1 KO similarly resulted in an accumulation of oxidized CE and TG lipids, which were suppressed by Fer-1 co-treatment (Fig. 5c). Having established that FSP1 inhibition results in the specific accumulation of oxidized TG and CE in cells with PUFA-TG-rich LDs, we next sought to identify the localization of this lipid peroxidation. Using the lipid peroxidation stain BODIPY-C11, we observed lipid peroxidation puncta that colocalized with the LD marker LipiBlue and were prevented by Fer-1 treatment (Fig. 5d,e).

**Extended Data Fig. 9:**
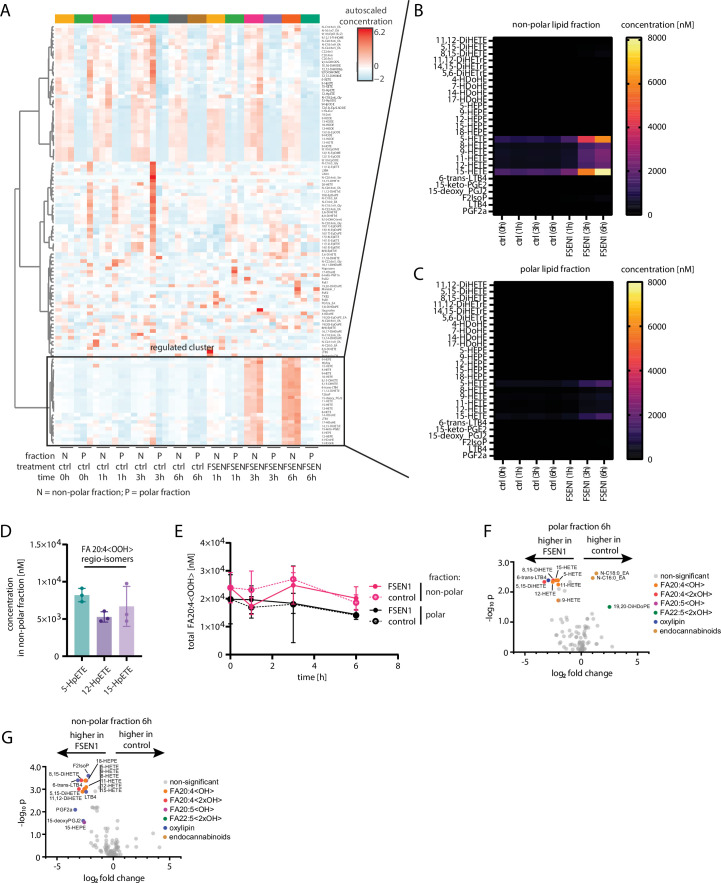
Absolute quantification of oxidized fatty acids in polar and non-polar lipid fractions of cells after induction of lipid droplet peroxidation. **A**: Hierarchical clustering of oxidized fatty acids in polar and non-polar lipid fractions as determined by targeted LC-MS/MS. U-2 OS cells were cultured in medium containing 200 µM FA 20:4 for 24 h followed by incubation with FSEN1 for 0, 1, 3, or 6 h. Quantities for individual oxidized fatty acids were autoscaled. **B**, **C**: Heatmap of oxidized fatty acids that specifically enrich in the polar (B) or non-polar (C) fraction during lipid droplet peroxidation induction. Quantities for individual oxidized fatty acids are concentrations. HETE = hydroxy-eicosa-tetra-en-oic acid, DiHETE = dihydroxy-eicosa-tetra-en-oic acid, DiHETrE = dihydroxy-eicosa-tri-en-oic acid, HDoHE = hydroxy-docosa-hexa-en-oic acid, HEPE = hydroxy-eicosa-penta-en-oic acid, LTB = leukotriene B, PGE = prostaglandin E, IsoP = isoprostane. **D**: Concentrations of individual hydroperoxide containing FA 20:4 regioisomers in the non-polar lipid fraction of cells cultivated for 24 h in 200 µM FA 20:4 containing medium followed by 6 h of FSEN1 treatment. (data are represented as mean ± SD, n = 3) **E**: All hydroperoxide containing FA 20:4 regio-isomers were summed up and the regulation of the overall levels of FA 20:4 hydroperoxides in polar and non-polar lipid fractions is displayed. (data are represented as mean ± SD, n = 3) **F**, **G**: U-2OS cells were cultured in medium supplemented with 200 µM FA 20:4 for 24 h, followed by incubation with FSEN for 6 h. Oxidized fatty acids in polar lipids (G) or non-polar lipids (H) were quantified via targeted LC-MS/MS after separation of lipid extracts into polar and non-polar lipid fractions, followed by basic hydrolysis. Data was autoscaled, thresholds for significant regulation are fold change > 2, statistical significance was assessed by two-sided t-test, values were deemed significant if p < 0.05 (FDR corrected). Source data

Pro-ferroptotic conditions are thought to induce the formation of phospholipid hydroperoxides, alcohols and ketones in the plasma membrane, but the accumulating oxidation products during LD peroxidation are not known. Current non-targeted epilipidomics workflows are incapable of identifying the exact structure of the various types of lipid modification and cannot provide quantitative information about the absolute levels of oxidized lipids. Therefore, to identify the main oxidized neutral lipids generated as a result of the loss of FSP1-mediated LD quality control, we extracted lipids from cells, separated the neutral lipid and polar lipid fractions using solid-phase extraction, and released esterified oxidized fatty acids via base hydrolysis, ultimately using a targeted approach that allows for absolute quantification of oxidized fatty acids (Extended Data Fig. 9a). Hierarchical clustering identified a cluster of oxidized fatty acids that specifically enriches in the neutral lipid fraction of cells with PUFA-TG-rich LDs (Extended Data Fig. 9a,b). This cluster contained almost exclusively alcohol-containing polyunsaturated fatty acids and identified various regioisomers of arachidonate-based alcohols (that is, hydroxy-eicosatetraenoic acids, HETE, FA20:4<OH>, <OH> = hydroxyl modification) as the main species accumulating as a result of FSP1 inhibition (Fig. 5f). Univariate statistics in polar and non-polar lipid fractions collected at different time points after FSP1 inhibition showed that the accumulation of arachidonate-based alcohols occurs only in the non-polar lipid fraction up to 3 h post FSP1 inhibition, but can also be detected in the polar lipid fraction after that (Fig. 5g,h and Extended Data Fig. 9f,g). Arachidonate-based alcohols are only detected in the phospholipid-containing polar lipid fraction at around 6 h post FSP1 inhibition, shortly before cell death (Fig. 5i). Interestingly, although arachidonate-based hydroperoxides (that is hydroperoxyl-eicosatetraenoic acids, HpETE, FA20:4<OOH>, <OOH> = hydroperoxyl modification) were highly enriched in the non-polar fraction of cells even at baseline (Extended Data Fig. 9d), these lipids did not accumulate or decrease during the observed time frame (Extended Data Fig. 9e). Several scenarios may explain why lipid alcohols, but not hydroperoxides, accumulate following FSP1 inhibition. One possibility is the presence of an enzymatic peroxidase system on LDs that selectively reduces lipid hydroperoxides to alcohols. Alternatively, the alkaline hydrolysis used during sample preparation may degrade hydroperoxides into alcohols, ketones, and epoxides^38^. This is further complicated by the lack of isotopically labelled internal standards for hydroperoxides, making their stability difficult to assess. It is also possible that the peroxidation environment in LDs inherently favours the formation of alcohols and other stable products. It remains unclear whether the observed alcohol accumulation reflects a biological process or an experimental artefact. These data indicate that PUFA-containing neutral lipids are highly reliant on FSP1 to prevent their peroxidation and that neutral lipid alcohols (and potentially other oxidized lipid derivatives that were not targetable with our approach) are the accumulating species driving LD peroxidation-mediated cell death.

### FSP1-mediated neutral lipid quality control prevents LD-initiated ferroptosis

**Fig. 6:**
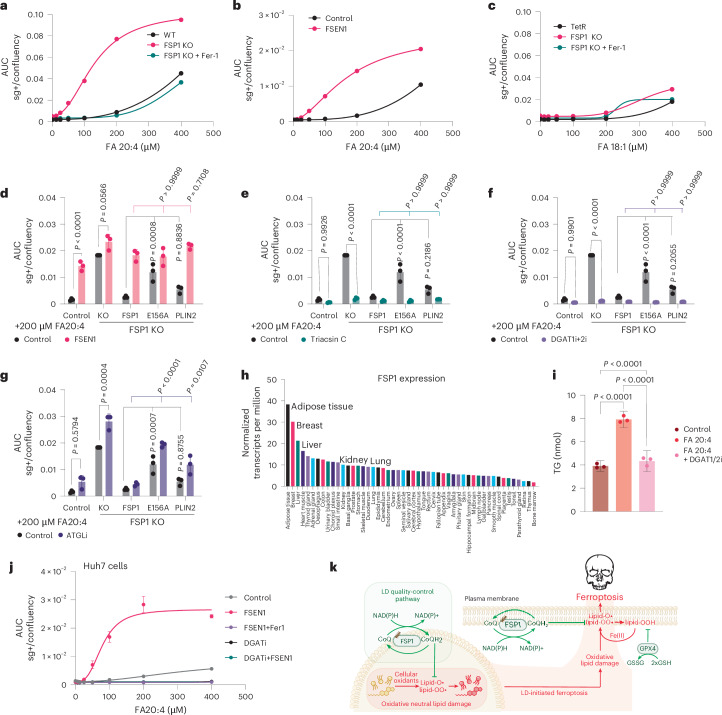
FSP1 suppresses LD-initiated ferroptosis. **a**–**c**, Cell death sensitivity to arachidonate (FA20:4) (**a**,**b**) or oleate (FA18:1) (**c**) treatment, assessed by time-resolved fluorescence microscopy (IncuCyte) with SYTOX Green over the course of 24 h. The number of SYTOX Green-positive objects per image equals the number of dead cells and is normalized to cell area per image expressed as confluency. sg+, SYTOX green-positive objects. Data are presented as mean ± s.e.m., *n* = 3). **d**–**g**, Cell death quantified for 200 µM FA20:4 after 24 h in the presence of FSP1 inhibitor (‘FSEN1’ indicates co-treatment with 5 μM FSEN1), DGAT1/2 inhibitors (co-treatment with 15 μM A-922500 and 10 μM PF-06424439), acyl-CoA synthetase inhibitor (‘triacsin C’ indicates co-treatment with 1 μg ml^−1^ triacsin C) or TG lipolysis inhibitor (‘ATGLi’ indicates co-treatment with 10 μM NG-497). Data are presented as mean ± s.d., *n* = 3. Statistical significance was assessed by one-way ANOVA. *P < 0.05; NS, not significant). **h**, Expression levels of FSP1 in human tissues. Data are derived from the Human Protein Atlas. **i**, Absolute quantification of TG levels in Huh7 cells through thin-layer chromatogram densitometry. Data are presented as mean ± s.e.m., *n* = 3. **j**, LD peroxidation-initiated ferroptosis in Huh7 cells by the exogenous addition of 200 µM FA20:4 after 24 h in the presence of FSP1 inhibitor (‘FSEN1’ indicates co-treatment with 5 μM FSEN1), DGATi (co-treatment with 15 μM A-922500 and 10 μM PF-06424439), radical-trapping antioxidant (‘Fer-1’ indicates co-treatment with 2 µM Fer-1). Data are presented as mean ± s.d., *n* = 3. Data were analysed using one-way ANOVA followed by Tukey’s post hoc test (two-sided), with adjustment for multiple comparisons. **k**, Schematic depicting the proposed mechanism of LD protection by FSP1. Source data

**Extended Data Fig. 10:**
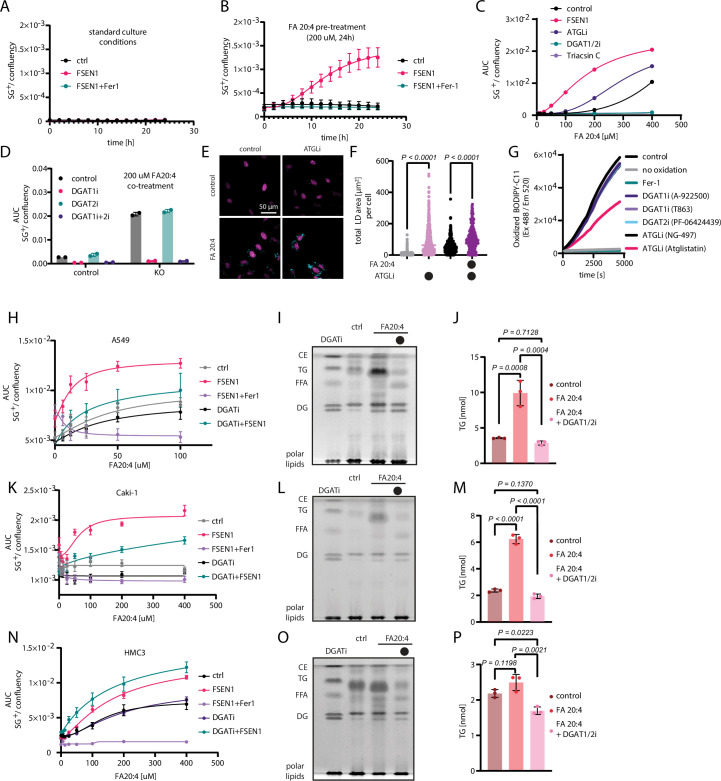
Non-polar lipid regulating compounds regulated lipid droplet initiated Ferroptosis and not by an off-target activity of lipid metabolism inhibitors. **A**–**D**: Cell death assessed by time resolved fluorescence microscopy (incucyte) with cell death indicator sytox green. (data in graphs A, B and D are represented as mean ± SD, n = 3) (data in graph C are represented as mean ± SEM, n = 3) **E**: Confocal microscopy of U-2 OS cultured in medium containing 200 µM FA20:4 in the presence or absence of ATGL inhibitor (ATGLi = cotreatment with 10 μM NG-497). **F**: Lipid droplets were imaged and lipid droplet area for each cell was quantified using Harmony data analysis software (Revvity). Each dot represents the lipid droplet area in an individual cell. (statistical significance is assessed by One-way ANOVA, * = p < 0.05, ns = not significant). **G**: Fluorescence enabled inhibited autooxidation in Egg-PC liposomes detecting no radical-trapping activity of utilized lipid metabolism inhibitors. (data are represented as mean, n = 3) **H**, **K**, **N**: Cell death in various cancer cell lines induced by lipid droplet initiated ferroptosis assessed by time resolved fluorescence microscopy (incucyte) with cell death indicator sytox green. (data are represented as mean ± SD, n = 3) **I**, **L**, **O**: Thin layer chromatogram displaying lipid changes in various cancer cell lines through addition of 200 µM FA20:4 for 24 h. **J**, **M**, **P**: Absolute quantification of triglyceride (TG) levels in various cancer cell lines via thin layer chromatogram densitometry. (data are represented as mean ± SD, n = 3) Data were analysed using one-way ANOVA followed by Tukey’s post hoc test (two-sided), with adjustment for multiple comparisons. Source data

Our cellular system provides a controlled model to investigate the cellular consequences of neutral lipid peroxidation. Arachidonate treatment was well tolerated by control cells, with minimal cell death observed at concentrations up to 200 µM (Fig. 6a). In contrast, FSP1 KO cells, and cells treated with FSEN1, exhibited heightened sensitivity to arachidonate, with cell death occurring at concentrations as low as 25 µM and substantial cell death observed at 200 µM (Fig. 6a,b and Extended Data Fig. 10a,b). This arachidonate-induced cell death in FSP1 KO cells was reversed by co-treatment with Fer-1 (Fig. 6a). Notably, although treatment with the monounsaturated fatty acid oleate increased LDs similar to arachidonate treatment (Fig. 4d–f), it did not induce appreciable cell death and there were no substantial differences between control and FSP1 KO cells (Fig. 6c). These findings identify a cell death that is induced by the peroxidation of PUFA-rich LDs, even in the absence of targeting the GSH-GPX4 pathway, highlighting the critical role of FSP1 in protecting cells from ferroptosis by maintaining neutral lipid quality control.

To investigate the importance of FSP1 activity and localization, we utilized our panel of cell lines expressing various FSP1 constructs. Expression of WT FSP1 strongly suppressed arachidonate-induced cell death in FSP1 KO cells, whereas this protective effect was abolished by the catalytically inactive E156A mutation (Fig. 6d). The LD-targeted FSP1 construct partially mitigated arachidonate-induced cell death in FSP1 KO cells, although not as effectively as WT FSP1, which localizes to multiple cellular compartments (Fig. 6d). The suppression of cell death by FSP1 was reversed by co-treatment with the FSP1 inhibitor FSEN1 (Fig. 6d), emphasizing the importance of FSP1 catalytic activity in protecting cells under these conditions. Inhibitors of acyl-CoA synthetases (for example, triacsin C) and DGAT1/2 effectively prevented arachidonate-induced cell death (Fig. 6e,f and Extended Data Fig. 10c,d), indicating that the cytotoxic effects of arachidonate in FSP1 KO cells are dependent on its activation and incorporation into TGs. Adipose triglyceride lipase (ATGL) inhibition increased LDs (Extended Data Fig. 10e,f), but did not prevent arachidonate-induced ferroptosis and slightly enhanced death in FSP1 KO cells (Fig. 6g and Extended Data Fig. 10c). Importantly, in contrast to the established radical-trapping antioxidant Fer-1, the utilized DGAT1/2 inhibitors and the human ATGL inhibitor (NG-497) had no radical-trapping antioxidant activity, supporting an on-target mechanism of action (Extended Data Fig. 10g). We noted that the mouse ATGL inhibitor atglistatin exhibited some radical-trapping antioxidant activity (Extended Data Fig. 10g). Although we did not use this inhibitor in our studies, we wanted to share the data as a cautionary note for the field.

FSP1 transcript levels are highest in LD-rich tissues such as adipose and liver, but considerable levels are also found in the kidney, lung and brain (Fig. 6h). Treatment with arachidonate resulted in increased TG levels in hepatoma (Huh7), lung adenocarcinoma (A549) and clear cell renal cell carcinoma (Caki-1) cells, but did not lead to considerable TG accumulation in a microglial cell line (HMC3) (Fig. 6i and Extended Data Fig. 6j,m,p). Strikingly, Huh7 cells (a liver hepatoma cell line) strongly relied on FSP1-mediated LD quality control, as inhibition of FSP1 under arachidonate treatment resulted in high rates of cell death at arachidonate concentrations as low as 50 µM, which was completely rescued by co-treatment with DGAT1/2 inhibitors (Fig. 6j). Other tested cell lines displayed cell death that was partially rescued by DGAT1/2 inhibition (A549, Caki-1), whereas the cell death observed in HMC3, a cell line that did not exhibit TG induction, was not rescued by DGAT1/2 inhibition. Thus, there is a cell type specificity that is dependent on the biogenesis of PUFA-rich LDs. Together, these results highlight the pivotal role of FSP1 in preventing ferroptosis induced by the accumulation of oxidatively damaged TGs in LDs, and its essentiality as a homeostatic mechanism for cell survival.

## Discussion

In this Article we have identifed FSP1 and CoQ10 as mediators of the first LD-specific lipid quality-control pathway (Fig. 6h), which prevents neutral lipid peroxidation and the initiation of ferroptosis from LDs. Remarkably, oxidized neutral lipid build-up and ferroptosis following FSP1 inhibition occur without GPX4 loss, revealing contexts where FSP1 is the primary suppressor. This expands the paradigm that FSP1 prevents peroxidation at multiple membranes, each representing distinct ferroptosis vulnerabilities.

LD-localized FSP1 reduces CoQ10 to generate local antioxidants that prevent neutral lipid peroxidation. CoQ10 often functions as a co-antioxidant with α-tocopherol, quenching tocopheroxyl radicals generated following the action of α-tocopherol as a radical-trapping antioxidant. However, LDs, which we find to lack detectable α-tocopherol or vitamin K in the conditions analysed in this study, appear to represent a context in which CoQ10 acts directly as the major radical scavenger. Other lipophilic antioxidants may contribute in specific tissues. How CoQ10 traffics to LDs remains an open question.

TG peroxidation may occur at packing defects in the LD monolayer^39,40^, which could expose TGs to reactive species, such as hydroxyl radicals and iron. Additionally, it is possible that oxidative damage initiates in the phospholipids of the LD monolayer and propagates inward to TG molecules in the LD core. The accumulation of oxidized neutral lipids probably impacts the structural and functional properties of LDs. Molecular dynamics simulations suggest that PUFA peroxidation within TG molecules increases their polarity, leading them to migrate from the neutral lipid core to the periphery of the LD, with the oxygen atoms protruding from between the LD phospholipid headgroups^41^. Structural changes resulting from oxidized fatty acids protruding between phospholipid headgroups would be expected to disrupt LD phospholipid packing, which is known to play important roles in LD protein targeting and association^1,4^. LDs would not be expected to rupture in the traditional manner of other organelles, because their lumen is made of neutral lipids rather than an aqueous compartment, however, the increased exposure of the hydrophobic oil core to the aqueous cytosol would probably disrupt LD morphology, alter protein association with LDs, and potentially facilitate the propagation of lipid peroxidation within LDs by enabling Fenton-type reactions with iron.

Our findings indicate that, in addition to the ER and lysosomes^23,42–44^, ferroptosis can also be triggered from LDs. LD-initiated ferroptosis required high PUFA-TG content and impaired FSP1 function. DGAT inhibitors, which block TG synthesis and LD biogenesis, effectively prevented ferroptosis in this context, underscoring the critical role of TGs and LDs in initiating ferroptosis under these conditions. Oxidation of LDs may contribute to ferroptosis under other conditions, such as ferroptosis induced by co-treatment with imidazole ketone erastin and the oxidized form of vitamin C^13^. Truncated forms and hydroxyl derivatives of TGs have been observed in dendritic cells in cancer and hypoxic trophoblast cells^41,45,46^, although their importance remains to be determined. The mechanisms by which lipid peroxidation propagates from intracellular organelles such as LDs to the plasma membrane remains unclear, but lipolysis is not required, as ATGL inhibition had no effect. It is possible that exposed TG peroxides at the LD surface could propagate oxidative damage by promoting iron oxidation, or LD phospholipid peroxides could be transferred to other membranes at membrane contact sites. The ER’s connection with LDs is notable, as LDs can grow through Ostwald ripening, a process in which the ER acts as a conduit for TG trafficking that enables neutral lipid exchange between LDs^47^. This suggests a potential pathway for the transmission of lipid peroxidation from LD neutral lipids to ER phospholipids, which could then spread through vesicular trafficking or phospholipid transfer at membrane contact sites.

The role of FSP1 in LD quality control has broad physiological and pathological implications. LDs are central in adipose and liver metabolism, and FSP1 is strongly expressed in brown adipose tissue^48^. LDs also accumulate under various pathological conditions. For instance, neurons efflux fatty acids (possibly lipid peroxides) for sequestration within astrocytic LDs as a protective mechanism^49–54^. Similarly, ferroptosis has been linked to liver diseases characterized by the accumulation of PUFA-containing TGs and LDs, such as metabolic dysfunction-associated fatty liver disease (MAFLD) and metabolic-associated steatohepatitis (MASH)^55–57^. These contexts underscore the potential biomedical importance of LD quality control in preventing ferroptosis-related damage.

In summary, we show that LD-localized FSP1 prevents neutral lipid oxidation and LD-initiated ferroptosis. LDs can be protective by sequestering PUFAs, but in the absence of FSP1, they become vulnerable sites of lipid peroxidation. These findings establish a unifying framework for LD lipid quality control and ferroptosis, highlighting the need for further study in physiology and disease.

## Methods

### LC–MS-based lipidomics and epilipidomics

Materials used for LC–MS were water (Fisher Scientific, W6-4), acetonitrile (Fisher Scientific, A955-4), 2-propanol (Supelco, 1.02781.4000), ammonium formate (Sigma-Aldrich, 70221-25G-F) and formic acid (Fisher Scientific, A117-50). Solvents for lipid extraction were *tert*-butyl methyl ether (Sigma-Aldrich, 34875) and methanol (Fisher Scientific, A456), spiked with 0.1% (wt/vol) 2,6-di-*tert*-butyl-4-methylphenol (Sigma-Aldrich, B1378). The lipid internal standard mixture was SPLASH LIPIDOMIX Mass Spec Standard (Avanti Research, 330707).

#### Lipid extraction for lipidomics and epilipidomics

Confluent culture plates (10 cm or 6 cm in diameter) were washed once with PBS and then scraped into 1 ml of ice-cold PBS. The cells were pelleted at 3,000*g* for 5 min at 4 C, the supernatant was removed, and the cell pellets were stored at −80 °C. Before extraction, the cell pellets were thawed at room temperature for 3 min and suspended in 50 µl of PBS. Internal standards (3 µl of SPLASH LIPIDOMIX per sample) dissolved in methanol were added directly to each cell suspension, then lipids were extracted by adding 1250 µl of *tert*-butyl methyl ether and 375 µl of methanol. The mixture was incubated on an orbital mixer for 1 h at room temperature (on a revolver shaker, 32 r.p.m.). To induce phase separation, 315 µl of water was added, and the mixture was incubated for 10 min at room temperature (on a revolver shaker, 32 r.p.m.). The samples were centrifuged at 15,000*g* at room temperature for 3 min, then the upper organic phase was collected and subsequently dried in vacuo (in an Eppendorf concentrator 5301).

#### Liquid chromatography

Dried lipid extracts were reconstituted in 150 µl of chloroform/methanol (2:1, vol/vol), and 20 µl of each extract was aliquoted in high-performance LC (HPLC) vials containing glass inserts. Pooled quality-control samples were generated by mixing equal volumes of each lipid extract followed by aliquotation in 20-µl aliquots. Aliquoted extracts and pooled quality controls were dried in vacuo (in an Eppendorf concentrator 5301) and redissolved in 20 µl of isopropanol for injection.

Lipids were separated by reversed-phase LC on a Vanquish Core system (Thermo Fisher Scientific) equipped with an Accucore C30 column (150 × 2.1 mm, 2.6 µm, 150 Å, Thermo Fisher Scientific). They were separated by gradient elution with solvent A (acetonitrile/water, 1:1, vol/vol) and B (isopropanol/acetonitrile/water, 85:10:5, vol/vol) both containing 5 mM ammonium formate and 0.1% (vol/vol) formic acid. Separation was performed at 50 °C at a flow rate of 0.3 ml min^−1^ using the following gradient: 0–15 min, 25–86% B (curve 5); 15–21 min, 86–100% B (curve 5); 21–32 min, 100% B isocratic; 32–32.1 min, 100–25% B (curve 5); followed by 6 min re-equilibration at 25% B.

#### Mass spectrometry

The reversed-phase LC set-up was coupled online to a Q Exactive Plus Hybrid Quadrupole Orbitrap mass spectrometer (Thermo Fisher Scientific) equipped with a heated electrospray ionization (HESI) probe. Mass spectra were acquired in positive and negative modes with the following ESI parameters: sheath gas, 40 l min^−1^; auxiliary gas, 10 l min^−1^; sweep gas, 1 l min^−1^; spray voltage, (+)3.5 kV (positive ion mode); (−)3.2 kV (negative ion mode); capillary temperature, 250 °C; S-lens RF level, 35; auxiliary gas heater temperature, 370 °C.

Data acquisition for lipid identification was performed in pooled quality-control samples by acquiring data in data-dependent acquisition mode (DDA). The DDA parameters featured a survey scan resolution of 140,000 (at *m/z* 200), AGC target of 1e6, maximum injection time of 100 ms in a scan range of *m/z* 240–1,200. Data-dependent MS/MS scans were acquired with a resolution of 17,500, AGC target of 1e5, maximum injection time of 60 ms, isolation window of 1.2 *m/z* and stepped normalized collision energies of 10, 20 and 30. A data-dependent MS2 was triggered (loop count of 15) when an AGC target of 2e2 was reached followed by a dynamic exclusion for 10 s. All isotopes and charge states >1 were excluded. All data were acquired in profile mode.

For deep lipidome profiling, iterative exclusion was performed using the IE omics R package^58^. This package generates a list for already fragmented precursors from a prior DDA run to be excluded from subsequent DDA runs, ensuring a higher number of unique MS/MS spectra for deep lipidome profiling. After the initial DDA analysis of a pooled quality-control sample, the pooled quality control was measured two more times, but excluding all previously fragmentated precursor ions. The samples were analysed both in positive and negative ion modes. The parameters for generating exclusion lists from previous runs were as follows: RT window of 0.3; noiseCount of 15; MZWindow of 0.02 and MaxRT of 36 min.

Data for lipid quantification in individual samples were acquired in full MS mode with the following parameters: scan resolution of 140,000 (at *m/z* 200), AGC target of 1e6 and maximum injection time of 100 ms in a scan range of *m/z* 240–1,200.

#### Lipid identification and quantification

Lipostar (version 2.0, Molecular Discovery), equipped with an in house-enerated structure database built in LipoStarDB manager, was used. This database features fatty acids with no information on double-bond regio- or stereoisomerism and covers glycerolipid, glycerophospholipid, sphingolipid and sterol ester lipid classes. The raw files were imported directly with Sample MS Signal Filter Signal Threshold = 1,000 for MS and Sample MS/MS Signal Filter Signal Threshold = 10. Automatic peak picking was performed with *m/z* tolerance = 5 ppm, chromatography filtering threshold = 0.97, MS filtering threshold = 0.97 and signal filtering threshold = 0. Peaks smoothing was performed using the Savitzky–Golay smoothing algorithm with window size = 3, degree = 2 and multi-pass iterations = 3. Isotopes were clustered using *m/z* tolerance = 5 ppm, RT tolerance = 0.25 min, abundance Dev = 40% and max charge = 1. Peak alignment between samples used *m/z* tolerance = 5 ppm and RT tolerance = 0.25 min. A gap filler with RT tolerance = 0.05 min and signal filtering threshold = 0 with an anti-spike filter was applied.

For lipid identification, an MS/MS-only filter was applied to keep only features with MS/MS spectra for identification. Triacylgylcerols, diacylglycerols and steryl esters were identified as [M + NH_4_]^+^ adducts. Lysophosphatidylcholines lysophosphatidylethanolamines, and acyl-, ether- and vinyl ether-PE, phosphatidylserines, phosphatidylinositols, ceramides and sphingomyelins were analysed as [M + H]^+^ adducts. Acyl-, ether- and vinyl ethers were identified as [M−H]^−^ adducts^59^. The following parameters were used for lipid identification: 5-ppm precursor ion mass tolerance and 20-ppm product ion mass tolerance. Automatic approval was performed to keep structures with a quality of 3–4 stars. Identifications were refined using manual curation and Kendrick mass defect analysis, and lipids that did not follow linear Kendrick mass defect–retention time correlations were excluded as false positives.

Quantification was performed by peak integration of the extracted ion chromatograms of single lipid adducts. Peak integration was manually curated and adjusted. Identified lipids were normalized to peak areas of added internal standards to decrease analytical variation, and eventually normalized to the protein concentrations of cell pellets after lipid extraction.

#### Statistical analysis of lipidomics data

MetaboAnalyst 5.0 (https://new.metaboanalyst.ca/home.xhtml) was used for statistical analysis and data transformation. Raw lipidomics data were imported into the ‘Statistical analysis [one factor]’ analysis pipeline. Data were transformed by ‘log transformation (base 10)’ and scaled by ‘auto scaling’ for statistical analysis. The transformed data and calculated statistics were exported as .csv files and plotted in GraphPad Prism 10.2.2 (GraphPad Software).

### Comprehensive epilipidomics (oxidized lipid analysis) for analysis of FSP1 mutants

#### Prediction of sample-specific oxidized lipidome data-dependent acquisition

For the semi-targeted identification of oxidized lipids from cell extracts we followed a protocol as published previously^57^. To narrow down the number of potential lipid peroxidation products, we chose a subset of lipids for in silico oxidation. This lipid subset consisted of the top ten lipids with the highest intensity of phosphatidylcholine (PC), plasmalogen phosphatidylcholine (P-PC), alkyl ether phosphatidylcholine (O-PC), phosphatidylethanolamine (PE), plasmalogen phosphatidylethanolamine (P-PE) and alkyl ether phosphatidylethanolamine (O-PE) and the top 20 lipids with the highest intensity of TG and CE lipid classes identified in each FSP1 mutant cell lipidome. We also incorporated the top ten lipids whose intensity correlates positively with sensitivity to cell death induced by RSL3 in each FSP1 mutant cell line. This led to a target list for the in silico oxidation of 176 lipids in total, represented by 77 phospholipids and 99 non-polar lipids.

In silico oxidation on this subset of rationally selected lipids was performed with LPPtiger2^34,35^ utilizing the following parameters: max modification site = 2, max total O = 3, max OH = 2, max keto = 1, max OOH = 1, max epoxy = 0. The predicted epilpidome was refined by the unmodified lipid list generated in the step described above. An inclusion list was generated featuring the [M+Na]^+^ adducts of these entries, as the generated fragments of sodiated oxidized lipid ions are most informative for oxidized lipid identification.

#### Liquid chromatography

Pooled quality-control samples were dissolved in 20 µl of isopropanol for injection. The lipids were separated by reversed-phase LC on a Vanquish Horizon system (Thermo Fisher Scientific) equipped with an Accucore C30 column (150 × 2.1 mm, 2.6 µm, 150 Å; Thermo Fisher Scientific). Lipids were separated by gradient elution with solvent A (acetonitrile/water, 1:1, vol/vol) and B (isopropanol/acetonitrile/water, 85:10:5, vol/vol) both containing 5 mM ammonium formate and 0.1% (vol/vol) formic acid. Separation was performed at 50 °C with a flow rate of 0.3 ml min^−1^ using the following gradient: 0–20 min, 10–80% B (curve 5); 20–24 min, 80–95% B (curve 5); 24–27 min, 95–100% B (curve 5); 27–32 min, 100% B isocratic; 32–32.1 min, 100–10% B (curve 5); followed by 7.9 min re-equilibration at 10% B.

#### Data-dependent acquisition settings to target the in silico oxidized epilipidome

The reversed-phase LC set-up was coupled online to an Orbitrap Exploris 240 Hybrid Quadrupole Orbitrap mass spectrometer (Thermo Fisher Scientific) equipped with a HESI probe. Mass spectra were acquired in positive and negative modes with the following ESI parameters: sheath gas, 40 arbitrary units (arb); auxiliary gas, 10 arb; sweep gas, 1 arb; spray voltage, (+)3.5 kV (positive ion mode); (–)2.5 kV (negative ion mode); ion transfer tube temperature, 300 °C; S-lens RF level, 35; vaporizer temperature, 370 °C. EASY-IC was set to run start.

Data acquisition for oxidized lipid identification was performed in quality-control samples by acquiring data in the data-dependent acquisition mode (DDA) with an inclusion list of predicted oxidized lipids. DDA parameters featured a survey scan resolution of 60,000 (at *m/z* 200), AGC target of 1e6, maximum injection time auto in a scan range of *m/z* 500–980 in negative ion mode (0–22 min) and *m/z* 480–1,200 in positive ion mode (22–40 min). Data-dependent MS/MS scans were acquired with a resolution of 60,000, normalized AGC target of 100%, maximum injection time of 200 ms, number of dependent scans, 6; isolation window, 1.5 *m/z*; first fixed mass, *m/z* 100; microscans, 2; normalized higher energy collision induced dissociation (HCD) collision energies, 22, 32, 43% in negative ion mode and 32, 43, 54% in positive ion mode. Dynamic exclusion was set to exclude precursors that were fragmented five times within 6 s were excluded from fragmentation for 5 s with a mass tolerance of ±5 ppm. All isotopes and charge states >1 were excluded. All data were acquired in profile mode.

#### Identification of oxidized lipids from the data-dependent acquisition dataset

MS/MS scans containing informative fragments were extracted using LPPtiger^35^ in lipid identification mode. The .raw files were converted to .mzML using MSConvertGUI from ProteoWizard 3.0.9134^60^. The .mzML files were uploaded to LPPtiger2, and MS/MS spectra containing oxidized lipid candidates were identified with the following settings: MS1 tolerance = 5 ppm, MSMS tolerance = 20 ppm, precursor intensity = 100, precursor mz window = *m/*z 480–1,100, Selection window = *m/z* 1.2 with a score filter > 60, isotope score > 80, rank score > 40 and considering peaks with intensity >1%.

To remove false-positive MS/MS candidates and improve identification confidence, the spectra were filtered manually, and MS/MS candidates that did not contain an ‘oxidized fatty acid specific fragment’ were removed. For oxTG and oxCE,n, ‘oxidized fatty acid specific fragment’ was defined as an [M + Na]^+^ or [M + H]^+^ adduct of an intact oxidized fatty acid (for example [FA18:2<OOH>+Na]^+^) or an oxidized fatty-acid fragment that after fragmentation retained at least one oxygen atom (for example, [FA18:2<OOH–H_2_O+H]^+^). MS/MS candidates containing fragments originating from oxidized fatty acids that do not retain additional oxygen atoms after fragmentation were removed (such as acid (for example, [FA18:2<OH>–H_2_O+H]^+^), as these spectra cannot be used to confidently determine the presence of a fatty acid-specific modification. For oxPC and oxPE, an oxidized fatty acid fragment was defined as an [M−H]^−^ adduct of an intact oxidized fatty acid (for example, [FA18:2<OOH>–H]^−^) or an oxidized fatty-acid fragment that after fragmentation retained at least one oxygen atom (for example, [FA18:2<OOH>–H_2_O–H]^−^). MS/MS candidates containing fragments originating from oxidized fatty acids that do not retain additional oxygen atoms after fragmentation were removed (such as acid (for example, [FA18:2<OH>–H_2_O–H]^−^) as these spectra cannot be used to confidently determine the presence of a fatty acid specific modification. Isomeric species were removed to result in a final list covering 82 *m/z* values representing oxidized lipids.

#### Relative quantification of oxidized lipids by parallel reaction monitoring

Lipids were dissolved in isopropanol and separated and ionized as described above for data-dependent acquisition.

Optimized data acquisition settings for the parallel reaction monitoring were as follows: isolation window = 1.5 *m/z*, resolution = 15,000, maximum IT = 100 ms, microscans = 1, featuring a single collision energy. Transitions were quantified with Skyline^59^ utilizing a 5-ppm mass accuracy on a centroided fragment signal. Confidence of quantification was improved by observing transitions of [M + Na]^+^ → [oxFA + Na]^+^ or [oxFA + H]^+^ depending on the targeted lipid.

The signal intensities of each oxidized lipid were normalized to isotopically labelled standards of their respective lipid class in the SPLASH LIPIDOMIX standard (Avanti Research, 330707) and finally normalized to the protein concentration in each cell pellet previously determined by a bicinchoninic acid protein assay kit (Thermo Fisher Scientific, PI23227).

#### Statistical analysis of epilipidomics data

MetaboAnalyst 5.0 (https://new.metaboanalyst.ca/home.xhtml) was used for statistical analysis and data transformation. Raw lipidomics data were imported into the ‘Statistical Analysis [one factor]’ analysis pipeline. The data were transformed by ‘Log transformation (base 10)’ and scaled by ‘auto scaling’ for statistical analysis. The transformed data and calculated statistics were exported as .csv files and plotted in GraphPad Prism 10.2.2 (GraphPad Software).

### Epilipidomics of oxidized non-polar lipids in the PUFA-induced LD damage model

#### Prediction of sample-specific oxidized lipidome data-dependent acquisition

For the untargeted identification of oxidized lipids from cell extracts, we followed a previously published protocol^59^. To narrow down the number of potential lipid peroxidation products, we chose a subset of lipids for in silico oxidation. This lipid subset consisted of the top 15 lipids with the highest intensity of TG and CE lipid classes in either U-2 OS WT cells treated with 200 µM arachidonic acid or U-2 OS TetR and U-2 OS FSP1 KO cells treated with 20 µM arachidonic acid. We also incorporated the lipids that were substantially downregulated (that is, log_2_(fold change) > 2, *P* < 0.05) by FSP1 inhibition via FSEN1 treatment in the arachidonic acid-treated conditions. This led to a target list for the in silico oxidation of 95 lipids, in total represented by 18 CE and 77 TG lipids.

In silico oxidation on this subset of rationally selected lipids was performed with LPPtiger^35^ with the following parameters: max modification site = 2, max total O = 3, max OH = 2, max keto = 1, max OOH = 1 and max epoxy = 0. The predicted epilipidome was refined by the unmodified lipid list generated in the step described above. An inclusion list was generated featuring the [M + Na]^+^ adducts of these entries, as the generated fragments of sodiated oxidized lipid ions are most informative for oxidized lipid identification.

#### Liquid chromatography

Dried lipid extracts were reconstituted in 60 µl of chloroform/methanol (2:1, vol/vol) and 10 µl of each extract was aliquoted in HPLC vials containing glass inserts. Quality-control samples were generated by mixing equal volumes of each lipid extract followed by aliquotation in 10-µl aliquots. Aliquoted extracts were dried in vacuo (in an Eppendorf concentrator 5301) and redissolved in 20 µl of isopropanol for injection, then 5 µl were loaded onto the column.

The lipids were separated by reversed-phase LC on a Vanquish Core system (Thermo Fisher Scientific) equipped with an Accucore C30 column (150 × 2.1 mm, 2.6 µm, 150 Å, Thermo Fisher Scientific). The lipids were separated by gradient elution with solvents A (acetonitrile/water, 1:1, vol/vol) and B (isopropanol/acetonitrile/water, 85:10:5, vol/vol), both containing 5 mM ammonium formate and 0.1% (vol/vol) formic acid. Separation was performed at 50 °C with a flow rate of 0.3 ml min^−1^ using the following gradient: 0–15 min, 25–86% B (curve 5); 15–21 min, 86–100% B (curve 5); 21–32 min, 100% B isocratic; 32–32.1 min, 100–25% B (curve 5); followed by 6 min re-equilibration at 25% B.

#### Data-dependent acquisition settings to target the in silico oxidized epilipidome

The reversed-phase LC set-up was coupled online to a QExactive Plus Hybrid Quadrupole Orbitrap mass spectrometer (Thermo Fisher Scientific) equipped with a HESI probe. Mass spectra were acquired in positive and negative modes with the following ESI parameters: sheath gas, 40 l min^−1^; auxiliary gas, 10 l min^−1^; sweep gas, 1 l min^−1^; spray voltage, (+)3.5 kV (positive ion mode; capillary temperature, 250 °C; S-lens RF level, 35; auxiliary gas heater temperature, 370 °C.

Data acquisition for oxidized lipid identification was performed in quality-control samples by acquiring data in data-dependent acquisition mode (DDA). The DDA parameters featured a survey scan resolution of 140,000 (at *m*/*z* 200), AGC target of 1e6 and a maximum injection time of 100 ms in a scan range of *m*/*z* 500–1,200. Data-dependent MS/MS scans were acquired with a resolution of 17,500, AGC target of 1e6, maximum injection time of 200 ms, loop count of 7, isolation window of 1.2 *m*/*z*, fixed fist mass of 100 and normalized collision energy of 30. A data-dependent MS/MS was triggered when an AGC target of 1e1 was reached followed by a dynamic exclusion for 3 s. All isotopes and charge states >1 were excluded. All data were acquired in profile mode.

#### Identification of oxidized lipids from the data-dependent acquisition dataset

MS/MS scans containing informative fragments were extracted using LPPtiger^35^ in lipid identification mode. The .raw files were converted to .mzML using MSConvertGUI from ProteoWizard 3.0.9134^60^. The .mzML files were uploaded to LPPtiger, and MS/MS spectra containing oxidized lipid candidates were identified with the following settings: MS1 tolerance = 5 ppm, MSMS tolerance = 20 ppm, precursor intensity = 100, precursor mz window = *m/*z 500–1,100, selection window = *m/z* 1.2 with a score filter > 60, isotope score > 80, rank score > 40 and considering peaks with intensity >1%.

To remove false-positive MS/MS candidates and improve identification confidence, the spectra were filtered manually, and MS/MS candidates that did not contain an oxidized fatty acid specific fragment were removed. An oxidized fatty acid specific fragment was defined as an [M + Na]^+^ or [M + H]^+^ adduct of an intact oxidized fatty acid (for example, [FA18:2<OOH> + Na]^+^) or an oxidized fatty acid fragment that at least retained one oxygen atom after fragmentation acid (for example, [FA18:2<OOH> – H_2_O + H]^+^). MS/MS candidates containing fragments originating from oxidized fatty acids that did not retain additional oxygen atoms after fragmentation were removed (such as acid (for example, [FA18:2<OH> – H_2_O + H]^+^), as these spectra cannot be used to confidently determine the presence of a fatty acid specific modification. All entries were sorted, and isomeric species were removed to result in a final inclusion list covering 16 *m/z* values representing oxidized lipids.

#### Relative quantification of oxidized lipids by parallel reaction monitoring

The lipids were dissolved in isopropanol and separated and ionized as described above for data-dependent acquisition.

The confidence of quantification was improved by observing transitions of [M + Na]^+^ → [oxFA + Na]^+^ or [oxFA + H]^+^ depending on the targeted lipid.

Optimized data acquisition settings for the parallel reaction monitoring were as follows: isolation width = 1.2 *m/z*, resolution = 17,500, maximum IT = 200 ms, microscans = 1, maximum IT = 200 ms featuring normalized collision energy = 30. Transitions were quantified with Skyline^61^ utilizing a 5-ppm mass accuracy on a centroided fragment signal. The signal intensities of each oxidized lipid were normalized to the isotopically labelled standards of their respective lipid class in the SPLASH LIPIDOMIX standard (Avanti Research, 330707).

#### Statistical analysis of epilipidomics data

MetaboAnalyst 5.0 (https://new.metaboanalyst.ca/home.xhtml) was used for statistical analysis and data transformation. Raw lipidomics data were imported into the ‘Statistical Analysis [one factor]’ analysis pipeline. The data were transformed by log transformation (base 10) and scaled by auto scaling for statistical analysis. The transformed data and calculated statistics were exported as .csv files and plotted in GraphPad Prism 10.2.2 (GraphPad Software).

### Relative quantification of redox active lipids

WT U-2 OS cells were treated with 200 µM oleate (Nu-Chek Prep) and 0.1% fatty acid=free BSA (Sigma-Aldrich, A3803). LDs were extracted as described below in the section ‘LD enrichment and flotation’.

Lipophilic radical-trapping antioxidants were extracted by the addition of 600 µl of methanol (Fisher Scientific, A456) + 0.1% (vol/vol) hydrochloric acid (Fisher Scientific, A144) and 600 µl hexane (Fisher Scientific, H302) to a 600-µl LD fraction. The samples were vortexed vigorously for 60 s at room temperature and centrifuged at 2,000*g* for 3 min at 4 °C. The upper hexane fraction was collected and transferred to a HPLC glass vial. The upper hexane phase was dried in vacuo (in a vacuum concentrator 5301, Eppendorf). For LC–MS analysis, the dried fraction was dissolved in 50 µl of isopropanol (Supelco, 1.02781.4000), and 10 µl were injected for analysis. Coenzyme Q10, Q9, Q8, vitamin K1 (phylloquinone), vitamin K2 (menaquinone-4) and (+)-α-tocopherol were analysed by parallel reaction monitoring.

The lipids were separated by reversed-phase LC on a Vanquish Core system (Thermo Fisher Scientific) equipped with an Accucore C30 column (150 × 2.1 mm, 2.6 µm, 150 Å, Thermo Fisher Scientific). Separation was carried out by gradient elution with solvent A (acetonitrile/water, 1:1, vol/vol) and B (isopropanol/acetonitrile/water, 85:10:5, vol/vol) both containing 5 mM ammonium formate and 0.1% (vol/vol) formic acid. Separation was performed at 50 °C with a flow rate of 0.3 ml min^−1^ using the following gradient: 0–15 min, 25–86% B (curve 5); 15–21 min, 86–100% B (curve 5); 21–32 min, 100% B isocratic; 32–32.1 min, 100–25% B (curve 5); followed by 6 min re-equilibration at 25% B.

The reversed-phase LC set-up was coupled online to a Q Exactive Plus Hybrid Quadrupole Orbitrap mass spectrometer (Thermo Fisher Scientific) equipped with a HESI probe. Mass spectra were acquired in positive and negative modes with the following ESI parameters: sheath gas, 40 l min^−1^; auxiliary gas, 10 l min^−1^; sweep gas, 1 l min^−1^; spray voltage, (+)3.5 kV (positive ion mode); (−)3.2 kV (negative ion mode); capillary temperature, 250 °C; S-lens RF level, 35; and auxiliary gas heater temperature, 370 °C.

Lipids were analysed by parallel reaction monitoring, and individual ions were isolated with an isolation window of 1.2 *m*/*z*. MS/MS spectra were acquired with an AGC target of 1e6 with a maxIT of 100 ms and a resolution of 17,500. The quantification of each lipid was optimized, and the final settings were CoQ10 (collision energy, 15; retention time, 22.4 min; precursor formula, C59H90O4; adduct, [M + NH_4_]^+^; product formula, C_10_H_12_O_4_; product *m*/*z*, 197.0808; product adduct, [M + H]^+^); CoQ10H_2_ (collision energy, 15; retention time, 22.1 min; precursor formula, C_59_H_92_O_4_; adduct, [M + NH_4_]^+^; product formula, C_10_H_12_O_4_; product *m*/*z*, 197.0808; product adduct, [M + H]^+^); CoQ9 (collision energy, 15; retention time, 21.4 min; precursor formula, C_54_H_82_O_4_; adduct, [M + NH_4_]^+^; product formula, C_10_H_12_O_4_; product *m*/*z*, 197.0808; product adduct, [M + H]^+^); CoQ9H_2_ (collision energy, 15; retention time, 21.1 min; precursor formula, C_54_H_84_O_4_; adduct, [M + NH_4_]^+^; product formula, C_10_H_12_O_4_; product *m*/*z*, 197.0808; product adduct, [M + H]^+^); CoQ8 (collision energy, 15; retention time, 20.2 min; precursor formula, C_49_H_74_O_4_; adduct, [M + NH_4_]^+^; product formula, C_10_H_12_O_4_; product *m*/*z*, 197.0808; product adduct, [M + H]^+^); vitamin K_1_ (collision energy, 25; retention time, 17.5 min; precursor formula, C_31_H_46_O_2_; adduct, [M + H]^+^; product formula, C_12_H_10_O_2_; product *m*/*z*, 187.0754; product adduct, [M + H]^+^); menaquinone-4 (collision energy, 25; retention time, 14.8 min; precursor formula, C_31_H_40_O_2_; adduct, [M + H]^+^; product formula, C_12_H_10_O_2_; product *m*/*z*, 187.0754; product adduct, [M + H]^+^); α-tocopherol (collision energy, 20; retention time, 13.2 min; precursor formula, C_29_H_50_O_2_; adduct, [M + H]^+^; product formula, C_10_H_12_O_2_; product *m*/*z*: 165.091; product adduct, [M + H]^+^).

The relative quantification of each analyte was performed by quantification of the transitions in the table above in Skyline^61^ utilizing a 5-ppm mass accuracy on a centroided signal.

### Cellular and in vitro assays

#### Cell culture

U-2 OS parental cells, Caki-1 cells and HMC3 cells were obtained from the UC Berkeley Cell Culture Facility. Huh7 cells were a kind gift from H. Ramage (University of Pennsylvania). U-2 OS, Caki-1 and Huh7 cells were cultured in DMEM with L-glutamine and without sodium pyruvate (Corning, 10-017-CM) and containing 10% fetal bovine serum (GemCell, 100-500). All cells were maintained at 37 °C and 5% CO_2_. A549 cells were a kind gift from S. Dixon’s laboratory (Stanford University) and were cultured in RPMI with L-glutamine (Corning, 10-040-CM) supplemented with 10% fetal bovine serum (GemCell, 100-500). HMC3 cells were cultured in EMEM (ATCC, 30-2003) with l-glutamine supplemented with 10% fetal bovine serum (GemCell, 100-500).

FSP1 mutant expression was induced in U-2 OS cells with 10 ng ml^−1^ doxocycline (Sigma-Aldrich, D9891) for 48 h before the respective experiments.

#### Immunodetection (western blotting) of FSP1

Cells were cultured in 10-cm culture dishes. Before collection, the cells were washed with 5 ml of PBS and scraped into 1 ml of PBS. The cells were pelleted by centrifugation at 3,000*g* for 3 min at 4 °C, and the supernatant was discarded. For lysis, the cells were resuspended in 100 µl of RIPA buffer (Pierce, 89901) with 1x protease inhibitor cocktail (Pierce, A32955) and sonicated by probe sonication for 30 s with 15% amplitude on a Branson 150 sonicator equipped with a sonication probe (Branson Ultrasonics, 4C15). Protein concentration was determined via a bicinchoninic acid protein assay kit (Thermo Fisher Scientific, PI23227).

For gel electrophoresis, the cell lysates were diluted with 1% sodium dodecyl sulfate (SDS) and 10–15 µg of protein were loaded on a 4–20% gradient gel (Bio-Rad, 4561094) and separated at 90 V for 10 min followed by 250 V for 20 min. The proteins were transferred to nitrocellulose membranes (Bio-Rad, 1704158) using the TransblotTurbo system (Bio-Rad) at mixed molecular-weight settings. The membranes were blocked with 5% milk in PBS containing 0.1% Tween-20 (Sigma-Aldrich, P7949) for 1 h at room temperature. The blots were washed three times with PBS containing 0.1% Tween-20. Primary antibodies were diluted 1:1,000 in 5% BSA (Fisher Scientific, BP9703) in PBS containing 0.1% Tween-20, and the blots were incubated with primary antibodies overnight at 4 °C. Next, blots were washed three times with PBS containing 0.1% Tween-20. Secondary antibodies were diluted 1:25,000 in 5% BSA in PBS containing 0.1% Tween-20. After washing three times with PBS containing 0.1% Tween-20, the blots were imaged on a Li-Cor imaging system (Odyssey).

#### Cell death analysis

Cells were seeded into 96-well plates (Corning, 3904) at 15% confluency in 10 ng ml^−1^ doxycycline hyclate (Sigma-Aldrich, D9891) for 48 h to induce FSP1 expression or 30% confluency for 24 h in WT cells before the respective experiments. LDs were induced by treating cells with 200 µM of the respective fatty acid (Nu-Chek Prep) and 0.1% fatty acid-free BSA (Sigma-Aldrich, A3803).

Ferroptosis initiators were dissolved in DMSO and mixed with the cell death marker SYTOX Green (Thermo Fisher, S34860) in standard culture medium to yield a 2x stock solution. A 100-µl volume of the 2x stock solution was added on top of 100 µl of medium in each well. Cell death propagation was followed by time-resolved fluorescence microscopy using an IncuCyte S3 imaging system (Essen Bioscience).

The fatty acids were dissolved in ethanol and mixed with SYTOX Green in standard culture medium to yield a 2x stock solution. To analyse the influence of lipid metabolism regulators, cells were co-treated with 5 µM FSEN1 (Cayman Chemicals, 38025), 1 µg ml^−1^ triacsin C (EnzoLifeSciences, BML-EI218), 10 µM NG-497 (Cayman Chemicals, 36886), 20 µM T-863 (Cayman Chemicals, 25807), 15 µM A-922500 (Cayman Chemicals, 10012708) or 10 µM PF-06424439 (Cayman Chemicals, 17680).

#### LD imaging and quantification

Cells were seeded into 96-well plates (Corning, 3904) at 30% confluency for 24 h in WT cells before imaging. LDs were induced by treating cells with 200 µM of the respective fatty acid (Nu-Chek Prep) and 0.1% fatty-acid-free BSA (Sigma-Aldrich, A3803). To block TG synthesis, DGAT inhibitors were added at the same time as the fatty acids. DGAT1 was inhibited by 15 µM A-922500 (Cayman Chemicals, 10012708) and DGAT2 was inhibited by 10 µM PF-06424439 (Cayman Chemicals, 17680).

For LD imaging, cells were stained with 1 μg ml^−1^ of BODIPY 493/503 (Invitrogen, D3922) for 25 min and Hoechst (Invitrogen, H3570), following the vendor’s recommendation. Medium was removed and replaced with pre-warmed phenol red-free medium (Cytiva, SH30284.02) supplemented with 10% fetal bovine serum (Gemini, 100-500).

BODIPY 493/503 was imaged on an Opera Phenix system (Revvity) with laser excitation at 488 nm, and emission was read at 500–550 nm. Hoechst was imaged using the standard 4′,6-diamidino-2-phenylindole (DAPI) filter sets.

LD number and area were quantified using Harmony data analysis software (Revvity).

#### LD enrichment and flotation

A total of 1.25 million cells were seeded in a p150 cell-culture dish and grown for 24 h. LD formation was induced with 200 µM oleate (Nu-Chek-Prep, U-46-A) and 0.1% fatty acid-free BSA (Sigma-Aldrich, A3803) for 24 h. Cells were collected by scraping into 3 ml of PBS and pelleted by centrifugation at 1,000*g* for 15 min at 4 °C. Cell pellets were resuspended in 2 ml of hypotonic lysis medium (20 mM Tris-HCl pH 7.4, 1 mM EDTA) with protease inhibitor cocktail (Thermo Fisher, A32955) and incubated for 10 min on ice. The cells were lysed by pushing the cell suspension through a 23-G needle (BD PrecisionGlide, 305145) eight times. The cell lysate was centrifuged at 1,000*g* for 10 min at 4 °C. For LD flotation, the supernatant was transferred to a 13.2-ml centrifuge tube (Beckman Coulter, 344059) and mixed with 60% sucrose (Fisher Scientific, S5-500) in hypotonic lysis medium to achieve a final concentration of 20% sucrose. This solution was overlaid with an ice-cold solution of 5% sucrose in hypotonic lysis medium followed by an ice-cold solution of hypotonic lysis medium without sucrose. Sucrose gradients were centrifuged at 28,000*g* for 30 min at 4 °C in an SW-41TI rotor (Beckman Coulter). The LD-containing buoyant fraction was collected as described previously^62^ and stored at −80 °C until analysis.

#### Absolute quantification of coenzyme Q10 in LDs

A 600-µl sample of the LD fraction was dried at room temperature for 5 min, then 2.5 pmol CoQ10-d6 (Cayman Chemicals, 30958) in isopropanol (Supelco, 1.02781.4000) was added, followed by vortexing. Coenzyme Q10 was extracted as described previously^63^ by the addition of 600 µl of methanol (Fisher Scientific, A456) + 0.1% (vol/vol) hydrochloric acid (Fisher Scientific, A144) and 600 µl of hexane (Fisher Scientific, H302). The samples were vortexed vigorously for 60 s at room temperature and centrifuged at 2,000*g* for 3 min at 4 °C. The upper hexane fraction was collected and transferred to a HPLC glass vial. The upper hexane phase was dried in vacuo (in a vacuum concentrator 5301, Eppendorf). For LC–MS analysis, the dried fraction was dissolved in 50 µl of isopropanol (Supelco, 1.02781.4000); a 10-µl volume was injected for analysis.

The lipids were separated by reversed-phase LC on a Vanquish Core system (Thermo Fisher Scientific) equipped with an Accucore C30 column (150 × 2.1 mm, 2.6 µm, 150 Å, Thermo Fisher Scientific). They were separated by gradient elution with solvent A (acetonitrile/water, 1:1, vol/vol) and B (isopropanol/acetonitrile/water, 85:10:5, vol/vol), both containing 5 mM ammonium formate and 0.1% (vol/vol) formic acid. Separation was performed at 50 °C with a flow rate of 0.3 ml min^−1^ using the following gradient: 0–15 min, 25–86% B (curve 5); 15–21 min, 86–100% B (curve 5); 21–32 min, 100% B isocratic; 32–32.1 min, 100–25% B (curve 5); followed by 6 min of re-equilibration at 25% B.

The reversed-phase LC set-up was coupled online to a Q Exactive Plus Hybrid Quadrupole Orbitrap mass spectrometer (Thermo Fisher Scientific) equipped with a HESI probe. Mass spectra were acquired in positive and negative modes with the following ESI parameters: sheath gas, 40 l min^−1^; auxiliary gas, 10 l min^−1^; sweep gas, 1 l min^−1^; spray voltage, (+)3.5 kV (positive ion mode); (–)3.2 kV (negative ion mode); capillary temperature, 250 °C; S-lens RF level, 35; and auxiliary gas heater temperature, 370 °C.

The quantification of each lipid was optimized with the following final settings: CoQ10 (collision energy, 15; retention time, 22.4 min; precursor formula, C_59_H_90_O_4_; adduct, [M + NH_4_]^+^; product formula, C_10_H_12_O_4_; product *m*/*z*, 197.0808; product adduct, [M + H]^+^); CoQ10H_2_ (collision energy, 15; retention time, 22.1 min; precursor formula, C_59_H_92_O_4_; adduct, [M + NH_4_]^+^; product formula, C_10_H_12_O_4_; product *m*/*z*, 197.0808; product adduct, [M + H]^+^); CoQ10-d_6_ (collision energy, 15; retention time, 22.4 min; precursor formula, C_59_H_84_D_6_O_4_; adduct, [M + NH_4_]^+^; product formula, C_10_H_6_D_6_O_4_; product *m*/*z*, 203.11904; product adduct, [M + H]^+^).

For absolute quantification, peak areas of CoQ10 and CoQ10H2 were divided by CoQ10-*d*6 and multiplied with the known concentration of CoQ10-*d*6.

#### Quantification of CoQ8 and CoQ10 in purified recombinant FSP1 preparations

CoQ8 and CoQ10 were quantified using targeted LC–MS/MS. The LC was performed on a Shimadzu SCL-40 high-performance LC system equipped with a YMC-Triart C18 column (50 ×2 mm, 3 μm) utilizing isocratic elution with methanol containing 2 mM ammonium formate with a flow rate of 0.4 ml min^−1^. CoQ forms were detected on a Shimadzu 8060NX triple quadrupole mass spectrometer via multiple reaction monitoring of the protonated and ammoniated adduct. The optimized transition settings were as follows: CoQ8 (adduct, [M + H]^+^; precursor *m*/*z*, 744.6; product *m*/*z*, 197.1; Q1, 20; collision energy, 22; Q3, 11); CoQ8 (adduct, [M + NH_4_]+; precursor *m*/*z*, 727.6; product *m*/*z*, 197.1; Q1, 26; collision energy, 28; Q3, 15); CoQ10 (adduct, [M + H]^+^; precursor *m*/*z*, 863.7; product *m*/*z*, 197.1; Q1, 30; collision energy, 38; Q3, 23); CoQ10 (adduct, [M + NH_4_]^+^; precursor *m*/*z*, 880.7; product *m*/*z*, 197.1; Q1, 20; collision energy, 21; Q3, 23).

Absolute quantification of CoQ8 and CoQ10 in recombinant FSP1 was performed through external calibration by generating calibration curves of authentic standards of CoQ8 (Avanti Research, 900151 P) and CoQ10 (Cayman Chemicals, 11506). CoQ forms were extracted from FSP1 recombinant protein preparations by using 5 μl of purified FSP1, followed by the addition of 200 μl of MeOH + 0.1% HCl and 200 μl of hexane. Samples were obtained by vortexing at room temperature for 60 s and centrifugation of the resulting suspensions at 2,000*g* for 3 min at room temperature to induce phase separation. The upper hexane fraction was dried in vacuo. Extracted CoQ was resuspended in 30 μl of MeOH containing 2 mM ammonium formate, and 5-μl samples were injected onto the column for analysis.

#### Thin-layer chromatography

The lipids were separated by thin-layer chromatography on HPTLC Silica gel 60 plates with a preconcentration zone (Supelco, 1.13748.0001). They were first dissolved in chloroform/methanol (2:1, vol/vol) and applied onto the TLC set-up with glass capillaries (CAMAG, 022.7729). Polar lipids were separated with triethylamine/chloroform/ethanol/water (5:5:5:1, vol/vol) and non-polar lipids were separated with hexane/diethyl ether/acetic acid (8:2:1, vol/vol) in a twin trough chamber (CAMAG, 022.5155) that was equilibrated with the eluent for 10 min before separation. The non-polar lipid standard mix comprised TG 18:1/18:1/18:1, free cholesterol, DG 18:1/0:0/18:1, CE 18:1, FA 18:1, Cer 18:1;O2/18:1. The polar lipid standard mix comprised TG 18:1/18:1/18:1, FA 18:1, PE 16:0/18:1, LPE 18:1, PC 16:0/18:1, SM 18:1;O2/18:1, LPC 18:0.

After separation, the plates were dried in air for 10 min, and the lipids were visualized by dipping the plates for 2 s into a solution of primuline (Sigma-Aldrich, 206865) (0.05%, wt/vol) in acetone/water (8:2, vol/vol). The plates were dried in air for 45 min and imaged on a ChemiDoc MP system (Bio-Rad) using blue epi light illumination and a 530/28 emission filter. Densitometric analysis was performed with Image Lab (version 5.2.1, Bio-Rad).

#### Solid-phase extraction for pre-analytical lipid separation

The lipids were separated into fatty acid, polar lipid and non-polar lipid fractions via solid-phase extraction on an aminopropyl STRATA NH2 column (500 mg, 55 μm, 70 Å, 8B-S009-HBJ, Phenomenex), as described previously^64^. The columns were equilibrated with 4 ml of hexane and lipid extracts were loaded onto the column in 400 μl of chloroform/methanol (2:1, vol/vol). Non-polar lipids were eluted with chloroform/isopropanol (2:1, vol/vol). Fatty acids were eluted with 8 ml of diethyl ether/acetic acid (100:2, vol/vol), and polar lipids were eluted with 4 ml of methanol. The collected fractions were dried in vacuo (in an Eppendorf concentrator 5301).

#### GC–FID for absolute fatty acid quantification in polar and non-polar lipid fractions

The fatty acid compositions of the polar lipid and non-polar lipid fractions were analysed at OmegaQuant (Sioux Falls, SD) via GC–FID. Esterified fatty acids were hydrolysed and methylated with an internal protocol, and the fatty acid methyl esters were separated on a GC2010 gas chromatograph set-up (Shimadzu) equipped with an SP2560 fused-silica capillary column (100 m, 0.25-mm internal diameter, 0.2-µm film thickness; Supelco). The fatty acids were identified based on the characteristic elution times of a fatty acid standard mixture (GLC OQ-A, Nu-Check Prep) and quantified based on external calibration.

#### Targeted LC–MS/MS analysis of esterified oxidized fatty acids in polar and non-polar lipid fractions

As shown previously, oxidized lipids are retained, during lipid fractionation, into polar and non-polar lipid fractions^65^. U-2 OS cells were treated with 200 μM FA20:4 for 24 h, followed by treatment with 5 μM FSEN1, and the cells were collected at 0, 1, 3 or 6 h post FSEN1 addition. The lipids were extracted and fractionated as described above.

Dried lipid extracts were resuspended in 5 µl of 0.2 mg ml^−1^ BHT/EDTA solution, 5 µl of standard surrogate mix, 25 µl of water and 475 µl of cold isopropanol chilled at −20 °C prior to addition. The samples were homogenized for 1 min, then kept at −80 °C for 1 h. The supernatant was then transferred into a 2-ml 96-well polypropylene plate; 100 µl of 0.6 M KOH solution was added to each well, and the plate was vortexed for 1 min. The samples were allowed to incubate at 60 °C for 30 min. After cooling to room temperature, the samples were diluted with 1 ml of 95:5 water/MeOH (vol/vol) with 0.1% acetic acid, and a 20-µl aliquot of 25% glacial acetic acid was added to neutralize the samples.

The analytes were isolated by solid-phase extraction on a 10-mg Oasis-HLB 96-well plate (Waters Corp). The plate wells were washed with 1 ml of ethyl acetate and 2 × 1 ml of methanol, followed by conditioning with 2 × 1 ml of 95:5 (vol/vol) water/methanol with 0.1% acetic acid. The samples were then transferred to the solid phase extraction (SPE) plate and allowed to elute by gravity. The plate wells were washed with 1 ml of 70:30 water/MeOH (vol/vol) + 0.1% acetic acid and then dried by vacuum at 7.5 inch of mercury (in Hg) for 10 min. The analytes were then eluted by gravity with 0.20 ml of MeOH + 1.0% acetic acid, followed by 1.20 ml of ethyl acetate in a 2-ml deep-well polypropylene 96-well plate containing 10 µl of 20% glycerol solution in MeOH in each well. The eluent was dried by centrifugal vacuum evaporation for ~3 h on a Genevac Ez2-Plus system (SP Scientific) using a low-boiling-point setting. The samples were reconstituted with 125 µl of 100 nM 1-cyclohexyl ureido, 3-dodecanoic acid (Sigma-Aldrich) and 1 phenyl ureido, 3-dodecanoic acid (gift from B. D. Hammock, University of California, Davis), at 100 nM in 50:50 MeOH:ACN. The plate was vortexed for 1 min and chilled for 15 min at –20 °C, after which extracts were transferred to a 0.2-µm polyvinylidene fluoride filter plate (Agilent Technologies), centrifuged for 3 min at 1,000*g* and 4 °C, and collected in a shallow 350-µl polypropylene 96-well plate, sealed with pre-slit silicon mats, and transferred to the instrument for LC–MS/MS analysis. Analyte separation by LC was performed on a Shimadzu Nexera X2 UHPLC set-up using a 2.1 x 150 mm, 1.7 µm Acquity BEH C18 column (Waters Corp). Separation was performed at a column temperature of 60 °C with gradient elution at a flow rate of 0.5 ml min^−1^. Solvent A was 0.1% acetic acid and solvent B was 90% acetonitrile and 10% isopropanol. The gradient was run as follows: 25% B from 0 to 0.25 min; 25–40% from 0.25 to 0.75 min; 40–42% from 0.75 to 1.5 min; 42–50% from 1.5 to 4.0 min; 50–57% from 4.25 to 6.75 min; 57–61% B from 6.75 to 8.0 min; 61–64% B from 8.0 to 10.5 min; 64–67% B from 10.5 to 11.0 min; 67–80% B from 11.0 to 13.0 min; 80–85% B from 13.0 to 13.3 min; 85–95% B from 13.3 to 13.85 min; 95–100% B from 13.85 to 14.0 min; isocratic at 100% B from 14.0 to 15.5 min; 100–25% B from 15.5 to 15.68 min; isocratic at 25% B from 15.68 to 17 min.

Mass spectrometry detection was performed on a 6500 QTRAP system (Sciex) with the following electrospray ionization parameters for negative (–)/positive (+) ionization: curtain gas = 20/20; IS = –4,500/4,500; TEM = 650/650; GS1 = 60/60; GS2 = 50/50; CAD = medium/medium; EP = –10/10; CXP = –13/12. Data acquisition parameters for all targeted analytes are provided in Supplementary Table 8. Analytes were quantified against a calibration curve bracketing reported concentrations using an isotope dilution and internal standard ratio-response approach. MS data were analysed using Multiquant 3.0.3 software (Sciex).

#### Recombinant FSP1 production

Expression vectors were transformed into LOBSTR-BL21 (DE3) cells (Kerafast, EC1002) and grown overnight in LB broth at 37 °C wile shaking (280 r.p.m.). Cultures were diluted 1:100 into 1 l of LB broth the next day and grown at 37 °C until reaching an optical density at 600 nm (OD_600_) of 0.6 (NanoDrop One, Thermo Fisher,13-400-518). Protein expression was induced with 0.7 mM IPTG, and cultures were incubated at 30 °C with shaking (280 r.p.m.) overnight. The cells were pelleted by centrifugation at 4,500 r.p.m. for 45 min at 4 °C and resuspended in 25 ml of lysis buffer (50 mM KH2PO4, 300 mM KCl, 10% glycerol, 30 mM imidazole, 1 mg ml^−1^ lysozyme, 1 mM phenylmethylsulfonyl fluoride (PMSF), pH 8.0). After 10 min of incubation at 37 °C with shaking (280 r.p.m.), the cells were lysed by passing through a microfluidizer (Microfluidics LM10) three times at 15,000 p.s.i. and clarified by ultracentrifugation at 50,000*g* for 30 min at 4 °C.

The clarified lysate was loaded onto an Econo-Pac column (Bio-Rad, 732-1010) containing 1 ml of HisPur Ni-NTA agarose resin (Thermo Fisher, 88221) equilibrated in wash buffer (50 mM KH_2_PO_4_, 300 mM KCl, 10% glycerol, 30 mM imidazole, pH 8.0). The column was washed four times with 2 ml wash buffer, and the proteins were eluted in 5 ml of elution buffer (same as the wash buffer but with 250 mM imidazole). The eluted proteins were buffer-exchanged into size exclusion chromatography (SEC) buffer (50 mM HEPES, 100 mM KCl, 2 mM dithiothreitol, pH 8.0, spiked with 15% glycerol and 10 mM dithiothreitol) using an Amicon Ultra-15 filter (Sigma-Aldrich, UFC910008) and concentrated to 3 ml.

Proteins were purified via size exclusion chromatography on a HiLoad 16/600 Superdex 75 Pg column (Sigma-Aldrich, GE28-9893-33) in SEC buffer using a GE Akta Pure FPLC system. Fractions (1.6 ml) were pooled based on SDS–PAGE purity (Coomassie-stained), concentrated to 2 mg ml^−1^, and snap-frozen in 50-μl aliquots. Protein concentration was determined using a Pierce BCA protein assay kit (Thermo Fisher, PI23227).

#### FSP1 activity assay—NADH reducing activity

The ability of recombinant WT FSP1 or the mutant FSP1(E156A) activity to consume NADH was tested in a 96-well format. Recombinant protein (25 nM) was mixed with the soluble CoQ10 derivative CoQ1 (Sigma-Aldrich, C7956) in PBS. Immediately before measurement, NADH (EMD Millipore, 481913) dissolved in PBS was added to result in a final concentration of 500 µM, then the reaction progress was followed using a TECAN Spark plate reader followed by measuring the absorbance of NADH at 340 nm every 15 s for 15 min. Data were plotted and analysed with GraphPad Prism (version 10.2.2).

#### FSP1 activity assay—CoQ reduction activity

The ability of recombinant WT FSP1 or the mutant FSP1(E156A) activity to reduce oxidized CoQ10 was tested in a 96-well format. Recombinant protein (25 nM) was mixed with NADH dissolved in PBS to result in a final concentration of 25 µM. Immediately before the measurement, CoQ1-coumarin (Cayman Chemicals, 29554) dissolved in PBS was added to the well, and reduction of CoQ1-coumarin was observed on a TECAN Spark Plate reader with monochromator excitation of 405/20 nm and monochromator emission readout at 475/10 nm with a gain of 95. Data were plotted and analysed with GraphPad Prism (version 10.2.2).

#### FSP1 activity assay—direct radical-trapping activity

We added 5 μl of a 6 mM solution of CoQ1 (Sigma-Aldrich, C7956) and 5 μl of a 1 mM solution of ammonium iron sulfate hexahydrate (Sigma-Aldrich, 215406) to a 96-well plate. A reaction mixture was premixed to contain LipiRadicalGreen (Diagnocine, FNK-FDV-0042) dissolved in DMSO, linolein hydroperoxides (Cayman Chemical, 89430) dissolved in ethanol, recombinant FSP1 protein and NADH dissolved in water. To achieve this, 550 μl of water, 3.4 μl of a 1 mM solution of LipiRadicalGreen, 28 μl of 500 µM linolein hydroperoxides in ethanol, 10.2 µl of a 10 μM solution of recombinant FSP1 and 28 µl of a 5 mM solution of NADH were mixed. The final concentrations of all the reactants were 500 nM LipiRadicalGreen, 50 μM linolein hydroperoxides, 50 µM iron, 150 nM FSP1, 200 µM NADH and 300 µM CoQ1. Of this reaction mixture, 90 μl was added to wells containing iron and CoQ1, and the fluorescence measurements were started immediately. LipiRadicalGreen activation was detected by fluorescence spectroscopy by excitation at 480/20 nm and emission at 540/10 nm with a gain of 100. The value of ∆LipiRadicalGreen was achieved by normalization with the fluorescence intensity at the reaction start in each well.

#### Artificial LD synthesis

Artificial lipid droplets were synthesized as previously published^66^. Briefly, 10 µmol TG 18:1/18:1/18:1 (Nu-Chek Prep, T-235), 0.95 µmol egg-PC (Avanti Research, 840051) and 18:1 DGS-NTA(Ni) (Avanti, 790404P) were each dissolved in 100 μl chloroform/methanol (2:1, vol/vol) and the solutions mixed. This resulted in a ratio of TG to egg-PC to 18:1DGS-NTA(Ni) of 10:0.95:0.05. The lipid mixture was dried in vacuo in HPLC glass vials (Eppendorf vaccuum concentrator 5301).

Dried lipids were resuspended in 1.25 ml of ethanol by vigorous vortexing for 60 s. To form artificial LDs, 1.25 ml of lipids dissolved in ethanol were added dropwise to a stirring solution of 3.75 ml of PBS containing 0.2% Tween-20 (Sigma-Aldrich, P7949). This solution was stirred for 4 h at room temperature in an open 10-ml glass beaker until all ethanol had evaporated, resulting in a solution containing 3.7 mM lipid.

#### Analysis of the supramolecular structure of artificial LDs and liposomes via Nile Red fluorescence

Artificial LDs or liposomes were diluted to 100 µM in 100 µl of PBS, and 2.5 µl of a 250 µM solution of Nile Red (Sigma-Aldrich, 19123) in ethanol was added and incubated for 15 min at room temperature. A TECAN Spark plate reader was used to read out Nile Red fluorescence by using monochromator excitation 530/10 nm with a gain of 100.

#### Quantification of artificial LD size and polydispersity by dynamic light scattering

Dynamic light scattering analysis was performed on a Brookhaven 90Plus Nanoparticle Analyzer. PBS was filtered three times through a 0.2 µm syringe filter (Pall Corporation, 4602) to remove particulate matter. A 3.5 mM stock solution of artificial LDs were diluted in filtered PBS 1:10,000 and analysed.

#### Recruitment analysis of recombinant FSP1 to artificial LDs

To probe the successful recruitment of FSP1 to artificial LDs, 2.5 mM artificial LDs were incubated with 250 µM FSP1 (artificial LD to FSP1 = 1,000:1) in a total volume of 250 µl for 1 h at room temperature. The artificial LD/FSP1 preparations were centrifuged at 17,000*g* for 5 min at room temperature to float the artificial LDs. The lower 175 µl were removed (unbound fraction) and the floating fraction was mixed with 175 µl of PBS. The artificial LD/FSP1 preparations were centrifuged again at 17,000*g* for 5 min at room temperature to float the artificial LDs. The lower 175 µl were collected (wash fraction), and 225 µl of PBS was added to the remaining liquid (artificial LD bound fraction). A 50-µl sample of each was mixed with SDS–PAGE Laemmli buffer (Bio-Rad, 1610747) and separated via gel electrophoresis (Bio-Rad, 4561094). Bands were visualized via silver staining (Thermo Fisher, 24600) and band densities were quantified using densitometry.

#### Liposome synthesis

Liposomes were generated by mixing 16.2 µmol egg-PC (Avanti Research, 840051) dissolved in chloroform/methanol (2:1, vol/vol) with 0.81 µmol 18:1 DGS-NTA(Ni) (Avanti, 790404 P) dissolved in chloroform/methanol (2:1, vol/vol) in a HPLC glass vial followed by drying in vacuo (Eppendorf vaccuum concentrator 5301). To form liposomes, 324 µl of PBS was added to the dried lipids and vortexed for 60 s at room temperature to yield a final concentration of 50 mM lipid. Unilamellar liposomes were generated by extrusion using a mini-extruder (Avanti, 610000) by 19 passes through a 0.1-µm membrane (Cytiva, 230300).

#### Recruitment analysis of recombinant FSP1 to liposomes

To probe the successful recruitment of FSP1 to liposomes, 10 mM liposomes were incubated with 1 µM FSP1 (liposomes to FSP1 = 1,000:1) in a total volume of 100 µl for 45 min at room temperature. In ultracentrifugation tubes (Beckman Coulter, 3437755) 30 µl of liposome/FSP1 solution was mixed with 30 µl of 60% sucrose in PBS to yield a final concentration of 30% sucrose. This was overlaid with 100 µl of 25% sucrose in PBS and subsequently with 20 µl of PBS. The tubes were centrifuged at 100,000*g* for 1 h at 4 °C with a TLA.100 rotor on a Beckmann Coulter Optima TL ultracentrifuge. Using gel loading tips, 155 µl of the bottom fraction was collected and discarded. The remaining floating fraction was mixed and collectedm then 16 µl of the floating fraction was mixed with SDS–PAGE Laemmli buffer (Bio-Rad, 1610747) following the vendor’s recommendations, and separated by gel electrophoresis (Bio-Rad, 4561094). The bands were visualized by silver staining (Thermo Fisher, 24600).

#### FENIX assay in artificial LDs and liposomes

BODIPY-C11 (Thermo Fisher Scientific, D3861) stock solutions were prepared in ethanol and stored at –80 °C until use. Artificial LD or liposome preparations were diluted to 2.5 mM and BODIPY-C11 was added to a final concentration of 2.5 µM (artificial LD to BODIPY-C11 = 1,000:1). A 40-µl sample of artificial LD/BODIPY-C11 or liposome/BODIPY-C11 solutions was added to a well in a 96-well plate, followed by 1 µl of a solution of coenzyme Q10 (Sigma-Aldrich, C9538) or other FSP1 substrates dissolved in ethanol. To this solution was added 10 µl of a FSP1 solution,10 µl of a NADH solution and 40 µl of PBS to achieve the desired concentration in a final reaction volume of 100 µl. This resulted in a final concentration of artificial LD/liposome (1 mM):BODIPY-C11 (1 µM):CoQ10:NADH. The oxidation initiator DTUN (Cayman Chemical, 32742) was dissolved in ethanol to yield a stock solution of 13.5 mM, and 1.5 µl of DTUN stock was added to a 100-µl reaction volume in each well.

A TECAN Spark plate reader was preheated to 37 °C, and the plates were shaken in the plate reader for 60 s using the ‘orbital’ shaking mode with an amplitude of 5. Reduced BODIPY-C11 was detected by fluorescence through monochromator excitation at 488/20 nm, and monochromator emission was detected at 590/10 nm at a gain of 120. Oxidized BODIPY-C11 was detected by fluorescence through excitation at 488/5 nm using a monochromator, and emission was detected at 520/10 nm at a gain of 120.

#### DepMap analysis

Gene essentiality correlation with lipid abundance across cell lines was performed on the DepMap portal (https://depmap.org/portal) as published previously^67^. A ‘custom analysis’ was set up and ‘Pearson correlation’ of the ‘gene = AIFM2 (FSP1, FLJ14497, AMID, PRG3)’ from the CRISPR (DepMap Public 24Q2+Score, Chronos) as ‘data slice’ with the dataset ‘Metabolomics’ of ‘all cell lines’ was calculated. The generated data were downloaded as a .csv file and plotted in GraphPad Prism version 10.2.2 (GraphPad Software).

#### Fluorescence microscopy for FSP1 construct subcellular localization

A total of 5,000 cells were seeded into a Lab-Tek II Chambered #1.5 German Coverglass system (Thermo Fisher, 155409) and induced with 10 ng ml^−1^ doxocycline for 24 h. The cells were treated with 200 µM FA18:1 in 0.1% BSA and 10 ng ml^−1^ doxycycline for another 24 h. LDs were stained with 200 nM LipiBlue (Dojindo, LD01-10) in phenol red-free medium supplemented with 10% fetal bovine serum for 1 h at 37 °C and 5% CO_2_. The cells were washed with warm phenol red-free medium and cell plasma membranes stained by incubating the cells in a 1:1,000 dilution of CellMask DeepRed (Thermo Fisher, C10046) in phenol red-free medium for 5 min. The staining medium was replaced with phenol red-free medium and imaging carried out on a Zeiss Axio Observer 7 system fitted with a x63 oil objective.

#### Fluorescence microscopy for LD colocalized lipid peroxidation

Glass-bottom microscopy dishes (35 mm, Mattek, P35G-1.5-14-C) were coated with poly-D-Lysine following the vendor’s recommendation. U-2 OS cells were seeded at 40% confluency and cultured for 16 h to adhere to the plates, then treated with 100 µM FA20:4 in 0.1% (wt/vol) fatty acid-free BSA (Sigma-Aldrich, 126575) for 24 h to induce LD formation without considerable cell death. After 24 h, the cells were treated with 5 µM FSEN1 in the presence or absence of 2 µM ferrostatin-1 for 4 h (that is, before considerable cell death sets in). Co-staining for LDs with LipiBlue (Dojindo, LD01-10) and lipid peroxidation with BODIPY-C11 (Invitrogen, D3861) was performed by washing cells once with warm PBS and co-treating the cells with 200 nM LipiBlue and 2 µM BODIPY-C11 in Hank’s Balanced Salt Solution (HBSS; Invitrogen, 14025-092) medium for 30 min at 37 °C and 5% CO_2_. After incubation, the staining medium was removed and replaced with warm HBSS. The cells were then imaged on a Zeiss LSM 880 FCS set-up at 37 °C and 5% CO_2_. The imaging settings were as follows: LipiBlue ex = 405 nm, em = 420–500 nm; reduced BODIPY-C11 ex = 561 nm, em = 580–650 nm; oxidized BODIPY-C11 ex = 488 nm, em = 500–550 nm.

Co-localization analysis of LipiBlue and BODIPY-C11 signals was performed with ImageJ/Fiji. First, the LipiBlue images were analysed to generate LipiBlue-positive regions of interest. LipiBlue-positive regions of interest were copied on images of reduced and oxidized BODIPY-C11, and the signal intensities in each channel were determined. Data were plotted in GraphPad Prism.

#### Sample size determination

No statistical methods were used to predetermine sample sizes, but our sample sizes were similar to those reported in previous publications^19,31^.

#### Assumptions for statistical tests

The data distribution was assumed to be normal, but this was not formally tested.

#### Blinding strategy

Samples for each mass spectrometry experiment were blinded and randomized before data acquisition and analysis and were unblinded following completion of data analysis.

#### Data exclusion

No data points were excluded from the presented analyses.

#### Materials availability

All unique/stable reagents generated in this study are available from the lead contact with a completed Materials Transfer Agreement.

### Reporting summary

Further information on research design is available in the Nature Portfolio Reporting Summary linked to this article.

## Online content

Any methods, additional references, Nature Portfolio reporting summaries, source data, extended data, supplementary information, acknowledgements, peer review information; details of author contributions and competing interests; and statements of data and code availability are available at 10.1038/s41556-025-01790-y.

## Supplementary information

Reporting Summary

Supplementary Tables Supplementary tables.

## Source data

Source Data Fig. 1 Source data for each presented plot.

Source Data Fig. 2 Source data for each presented plot.

Source Data Fig. 3 Source data for each presented plot.

Source Data Fig. 4 Source data for each presented plot.

Source Data Fig. 5 Source data for each presented plot.

Source Data Fig. 6 Source data for each presented plot.

Source Data Extended Data Fig. 1 Uncropped western blots.

Source Data Extended Data Fig. 1 Source data for each presented plot.

Source Data Extended Data Fig. 2 Uncropped western blots.

Source Data Extended Data Fig. 3 Source data for each presented plot.

Source Data Extended Data Fig. 4 Uncropped western blots.

Source Data Extended Data Fig. 4 Source data for each presented plot.

Source Data Extended Data Fig. 5 Source data for each presented plot.

Source Data Extended Data Fig. 6 Source data for each presented plot.

Source Data Extended Data Fig. 7 Source data for each presented plot.

Source Data Extended Data Fig. 8 Source data for each presented plot.

Source Data Extended Data Fig. 9 Source data for each presented plot.

Source Data Extended Data Fig. 10 Source data for each presented plot.

## Data Availability

Processed data are available as a supplement to this manuscript. Source data are provided with this paper.

## References

[1] Mathiowetz, A. J. & Olzmann, J. A. Lipid droplets and cellular lipid flux. *Nat. Cell Biol.* **26**, 331–345 (2024). 38454048 10.1038/s41556-024-01364-4 PMC11228001

[2] Olzmann, J. A. & Carvalho, P. Dynamics and functions of lipid droplets. *Nat. Rev. Mol. Cell Biol.* **20**, 137–155 (2019). 30523332 10.1038/s41580-018-0085-z PMC6746329

[3] Walther, T. C., Kim, S., Arlt, H., Voth, G. A. & Farese, R. V. Structure and function of lipid droplet assembly complexes. *Curr. Opin. Struct. Biol.* **80**, 102606 (2023). 37150040 10.1016/j.sbi.2023.102606 PMC10853036

[4] Olarte, M.-J., Swanson, J. M. J., Walther, T. C. & Farese, R. V. The CYTOLD and ERTOLD pathways for lipid droplet–protein targeting. *Trends Biochem. Sci.* **47**, 39–51 (2022). 34583871 10.1016/j.tibs.2021.08.007 PMC8688270

[5] Nguyen, T. B. et al. DGAT1-dependent lipid droplet biogenesis protects mitochondrial function during starvation-induced autophagy. *Dev. Cell* **42**, 9–21.e5 (2017). 28697336 10.1016/j.devcel.2017.06.003 PMC5553613

[6] Chitraju, C. et al. Triglyceride synthesis by DGAT1 protects adipocytes from lipid-induced ER stress during lipolysis. *Cell Metab.* **26**, 407–418.e3 (2017). 28768178 10.1016/j.cmet.2017.07.012 PMC6195226

[7] Hegde, R. S. & Ploegh, H. L. Quality and quantity control at the endoplasmic reticulum. *Curr. Opin. Cell Biol.* **22**, 437–446 (2010). 20570125 10.1016/j.ceb.2010.05.005 PMC2929805

[8] Sancar, A., Lindsey-Boltz, L. A., Ünsal-Kaçmaz, K. & Linn, S. Molecular mechanisms of mammalian DNA repair and the DNA damage checkpoints. *Annu. Rev. Biochem.* **73**, 39–85 (2004). 15189136 10.1146/annurev.biochem.73.011303.073723

[9] Li, Z., Lange, M., Dixon, S. J. & Olzmann, J. A. Lipid quality control and ferroptosis: from concept to mechanism. *Annu. Rev. Biochem.* **93**, 499–528 (2024). 37963395 10.1146/annurev-biochem-052521-033527 PMC11091000

[10] Lee, H. et al. Cell cycle arrest induces lipid droplet formation and confers ferroptosis resistance. *Nat. Commun.* **15**, 79 (2024). 38167301 10.1038/s41467-023-44412-7 PMC10761718

[11] Dierge, E. et al. Peroxidation of *n*–3 and *n*–6 polyunsaturated fatty acids in the acidic tumor environment leads to ferroptosis-mediated anticancer effects. *Cell Metab.* **33**, 1701–1715.e5 (2021). 34118189 10.1016/j.cmet.2021.05.016

[12] Minami, J. K. et al. CDKN2A deletion remodels lipid metabolism to prime glioblastoma for ferroptosis. *Cancer Cell* **41**, 1048–1060.e9 (2023). 37236196 10.1016/j.ccell.2023.05.001 PMC10330677

[13] Ferrada, L., Barahona, M. J., Vera, M., Stockwell, B. R. & Nualart, F. Dehydroascorbic acid sensitizes cancer cells to system xc- inhibition-induced ferroptosis by promoting lipid droplet peroxidation. *Cell Death Dis.* **14**, 637 (2023). 37752118 10.1038/s41419-023-06153-9 PMC10522586

[14] Lorito, N. et al. FADS1/2 control lipid metabolism and ferroptosis susceptibility in triple-negative breast cancer. *EMBO Mol. Med.* **16**, 1533–1559 (2024). 38926633 10.1038/s44321-024-00090-6 PMC11251055

[15] Dixon, S. J. & Olzmann, J. A. The cell biology of ferroptosis. *Nat. Rev. Mol. Cell Biol.* **25**, 424–442 (2024). 38366038 10.1038/s41580-024-00703-5 PMC12187608

[16] Yang, W. S. et al. Regulation of ferroptotic cancer cell death by GPX4. *Cell* **156**, 317–331 (2014). 24439385 10.1016/j.cell.2013.12.010 PMC4076414

[17] Doll, S. et al. FSP1 is a glutathione-independent ferroptosis suppressor. *Nature* **575**, 693–698 (2019). 31634899 10.1038/s41586-019-1707-0

[18] Bersuker, K. et al. The CoQ oxidoreductase FSP1 acts parallel to GPX4 to inhibit ferroptosis. *Nature* **575**, 688–692 (2019). 31634900 10.1038/s41586-019-1705-2 PMC6883167

[19] Mishima, E. et al. A non-canonical vitamin K cycle is a potent ferroptosis suppressor. *Nature* **608**, 778–783 (2022). 35922516 10.1038/s41586-022-05022-3 PMC9402432

[20] Jin, D.-Y. et al. A genome-wide CRISPR-Cas9 knockout screen identifies FSP1 as the warfarin-resistant vitamin K reductase. *Nat. Commun.* **14**, 828 (2023). 36788244 10.1038/s41467-023-36446-8 PMC9929328

[21] Pope, L. E. & Dixon, S. J. Regulation of ferroptosis by lipid metabolism. *Trends Cell Biol.* **33**, 1077–1087 (2023). 37407304 10.1016/j.tcb.2023.05.003 PMC10733748

[22] Rodencal, J. & Dixon, S. J. A tale of two lipids: lipid unsaturation commands ferroptosis sensitivity. *Proteomics* **23**, 2100308 (2023). 10.1002/pmic.202100308 36398995

[23] Magtanong, L. et al. Exogenous monounsaturated fatty acids promote a ferroptosis-resistant cell state. *Cell Chem. Biol.* **26**, 420–432.e9 (2019). 30686757 10.1016/j.chembiol.2018.11.016 PMC6430697

[24] Doll, S. et al. ACSL4 dictates ferroptosis sensitivity by shaping cellular lipid composition. *Nat. Chem. Biol.* **13**, 91–98 (2017). 27842070 10.1038/nchembio.2239 PMC5610546

[25] Dixon, S. J. et al. Human haploid cell genetics reveals roles for lipid metabolism genes in nonapoptotic cell death. *ACS Chem. Biol.* **10**, 1604–1609 (2015). 25965523 10.1021/acschembio.5b00245 PMC4509420

[26] Kagan, V. E. et al. Oxidized arachidonic and adrenic PEs navigate cells to ferroptosis. *Nat. Chem. Biol.* **13**, 81–90 (2017). 27842066 10.1038/nchembio.2238 PMC5506843

[27] Liang, D. et al. Ferroptosis surveillance independent of GPX4 and differentially regulated by sex hormones. *Cell* **186**, 2748–2764.e22 (2023). 37267948 10.1016/j.cell.2023.05.003 PMC10330611

[28] Rodencal, J. et al. Sensitization of cancer cells to ferroptosis coincident with cell cycle arrest. *Cell Chem. Biol.* **31**, 234–248.e13 (2024). 37963466 10.1016/j.chembiol.2023.10.011 PMC10925838

[29] Phadnis, V. V. et al. MMD collaborates with ACSL4 and MBOAT7 to promote polyunsaturated phosphatidylinositol remodeling and susceptibility to ferroptosis. *Cell Rep.* **42**, 113023 (2023). 37691145 10.1016/j.celrep.2023.113023 PMC10591818

[30] Lange, M., Wagner, P. V. & Fedorova, M. Lipid composition dictates the rate of lipid peroxidation in artificial lipid droplets. *Free Radic. Res.* **55**, 469–480 (2021). 33866899 10.1080/10715762.2021.1898603

[31] Freitas, F. P. et al. 7-Dehydrocholesterol is an endogenous suppressor of ferroptosis. *Nature* **626**, 401–410 (2024). 38297129 10.1038/s41586-023-06878-9

[32] Li, Y. et al. 7-Dehydrocholesterol dictates ferroptosis sensitivity. *Nature* **626**, 411–418 (2024). 38297130 10.1038/s41586-023-06983-9 PMC11298758

[33] Yamada, N. et al. Inhibition of 7-dehydrocholesterol reductase prevents hepatic ferroptosis under an active state of sterol synthesis. *Nat. Commun.* **15**, 2195 (2024). 38472233 10.1038/s41467-024-46386-6 PMC10933264

[34] Criscuolo, A. et al. Analytical and computational workflow for in-depth analysis of oxidized complex lipids in blood plasma. *Nat. Commun.* **13**, 6547 (2022). 36319635 10.1038/s41467-022-33225-9 PMC9626469

[35] Ni, Z., Angelidou, G., Hoffmann, R. & Fedorova, M. LPPtiger software for lipidome-specific prediction and identification of oxidized phospholipids from LC-MS datasets. *Sci. Rep.* **7**, 15138 (2017). 29123162 10.1038/s41598-017-15363-z PMC5680299

[36] Shah, R., Farmer, L. A., Zilka, O., Van Kessel, A. T. M. & Pratt, D. A. Beyond DPPH: use of fluorescence-enabled inhibited autoxidation to predict oxidative cell death rescue. *Cell Chem. Biol.* **26**, 1594–1607.e7 (2019). 31564533 10.1016/j.chembiol.2019.09.007

[37] Mishima, E. et al. Drugs repurposed as antiferroptosis agents suppress organ damage, including AKI, by functioning as lipid peroxyl radical scavengers. *J. Am. Soc. Nephrol.* **31**, 280–296 (2020). 31767624 10.1681/ASN.2019060570 PMC7003311

[38] Gardner, H. W., Simpson, T. D. & Hamberg, M. Mechanism of linoleic acid hydroperoxide reaction with alkali. *Lipids* **31**, 1023–1028 (1996). 8898300 10.1007/BF02522458

[39] Bacle, A., Gautier, R., Jackson, C. L., Fuchs, P. F. J. & Vanni, S. Interdigitation between triglycerides and lipids modulates surface properties of lipid droplets. *Biophys. J.* **112**, 1417–1430 (2017). 28402884 10.1016/j.bpj.2017.02.032 PMC5390054

[40] Kim, S., Swanson, J. M. J. & Voth, G. A. Computational studies of lipid droplets. *J. Phys. Chem. B* **126**, 2145–2154 (2022). 35263109 10.1021/acs.jpcb.2c00292 PMC8957551

[41] Mohammadyani, D. et al. Molecular speciation and dynamics of oxidized triacylglycerols in lipid droplets: mass spectrometry and coarse-grained simulations. *Free Radic. Biol. Med* **76**, 53–60 (2014). 25110833 10.1016/j.freeradbiomed.2014.07.042 PMC4276254

[42] Von Krusenstiern, A. N. et al. Identification of essential sites of lipid peroxidation in ferroptosis. *Nat. Chem. Biol.* **19**, 719–730 (2023). 36747055 10.1038/s41589-022-01249-3 PMC10238648

[43] Gaschler, M. M. et al. Determination of the subcellular localization and mechanism of action of ferrostatins in suppressing ferroptosis. *ACS Chem. Biol.* **13**, 1013–1020 (2018). 29512999 10.1021/acschembio.8b00199 PMC5960802

[44] Cañeque, T. et al. Activation of lysosomal iron triggers ferroptosis in cancer. *Nature* **642**, 492–500 (2025). 40335696 10.1038/s41586-025-08974-4 PMC12158755

[45] Cao, W. et al. Oxidized lipids block antigen cross-presentation by dendritic cells in cancer. *J. Immunol.* **192**, 2920–2931 (2014). 24554775 10.4049/jimmunol.1302801 PMC3998104

[46] Tyurin, V. A., Cao, W., Tyurina, Y. Y., Gabrilovich, D. I. & Kagan, V. E. Mass-spectrometric characterization of peroxidized and hydrolyzed lipids in plasma and dendritic cells of tumor-bearing animals. *Biochem. Biophys. Res. Commun.* **413**, 149–153 (2011). 21872574 10.1016/j.bbrc.2011.08.074 PMC3356782

[47] Salo, V. T. et al. Seipin facilitates triglyceride flow to lipid droplet and counteracts droplet ripening via endoplasmic reticulum contact. *Dev. Cell* **50**, 478–493.e9 (2019). 31178403 10.1016/j.devcel.2019.05.016

[48] Nguyen, H. P. et al. Aifm2, a NADH oxidase, supports robust glycolysis and is required for cold- and diet-induced thermogenesis. *Mol. Cell* **77**, 600–617.e4 (2020). 31952989 10.1016/j.molcel.2019.12.002 PMC7031813

[49] Liu, L. et al. Glial lipid droplets and ROS induced by mitochondrial defects promote neurodegeneration. *Cell* **160**, 177–190 (2015). 25594180 10.1016/j.cell.2014.12.019 PMC4377295

[50] Liu, L., MacKenzie, K. R., Putluri, N., Maletić-Savatić, M. & Bellen, H. J. The glia-neuron lactate shuttle and elevated ROS promote lipid synthesis in neurons and lipid droplet accumulation in glia via APOE/D. *Cell Metab.* **26**, 719–737.e6 (2017). 28965825 10.1016/j.cmet.2017.08.024 PMC5677551

[51] Moulton, M. J. et al. Neuronal ROS-induced glial lipid droplet formation is altered by loss of Alzheimer’s disease-associated genes. *Proc. Natl Acad. Sci. USA* **118**, e2112095118 (2021). 34949639 10.1073/pnas.2112095118 PMC8719885

[52] Ioannou, M. S. et al. Neuron–astrocyte metabolic coupling protects against activity-induced fatty acid toxicity. *Cell* **177**, 1522–1535.e14 (2019). 31130380 10.1016/j.cell.2019.04.001

[53] Ralhan, I. et al. Autolysosomal exocytosis of lipids protect neurons from ferroptosis. *J. Cell Biol.* **222**, e202207130 (2023). 37036445 10.1083/jcb.202207130 PMC10098143

[54] Bailey, A. P. et al. Antioxidant role for lipid droplets in a stem cell niche of drosophila. *Cell* **163**, 340–353 (2015). 26451484 10.1016/j.cell.2015.09.020 PMC4601084

[55] Chen, J., Li, X., Ge, C., Min, J. & Wang, F. The multifaceted role of ferroptosis in liver disease. *Cell Death Differ.* **29**, 467–480 (2022). 35075250 10.1038/s41418-022-00941-0 PMC8901678

[56] Johnson, S. M. et al. PNPLA3 is a triglyceride lipase that mobilizes polyunsaturated fatty acids to facilitate hepatic secretion of large-sized very low-density lipoprotein. *Nat. Commun.* **15**, 4847 (2024). 38844467 10.1038/s41467-024-49224-x PMC11156938

[57] Luukkonen, P. K. et al. Human PNPLA3-I148M variant increases hepatic retention of polyunsaturated fatty acids. *JCI Insight* **4**, e127902 (2019). 31434800 10.1172/jci.insight.127902 PMC6777808

[58] Koelmel, J. P. et al. Expanding lipidome coverage using LC-MS/MS data-dependent acquisition with automated exclusion list generation. *J. Am. Soc. Mass. Spectrom.* **28**, 908–917 (2017). 28265968 10.1007/s13361-017-1608-0 PMC5408749

[59] Lange, M. et al. AdipoAtlas: a reference lipidome for human white adipose tissue. *Cell Rep. Med.* **2**, 100407 (2021). 34755127 10.1016/j.xcrm.2021.100407 PMC8561168

[60] Chambers, M. C. et al. A cross-platform toolkit for mass spectrometry and proteomics. *Nat. Biotechnol.* **30**, 918–920 (2012). 23051804 10.1038/nbt.2377 PMC3471674

[61] Adams, K. J. et al. Skyline for small molecules: a unifying software package for quantitative metabolomics. *J. Proteome Res* **19**, 1447–1458 (2020). 31984744 10.1021/acs.jproteome.9b00640 PMC7127945

[62] Peterson, C. W. H., Deol, K. K., To, M. & Olzmann, J. A. Optimized protocol for the identification of lipid droplet proteomes using proximity labeling proteomics in cultured human cells. *STAR Protoc.* **2**, 100579 (2021). 34151299 10.1016/j.xpro.2021.100579 PMC8190507

[63] Burger, N. et al. A sensitive mass spectrometric assay for mitochondrial CoQ pool redox state in vivo. *Free Radic. Biol. Med* **147**, 37–47 (2020). 31811922 10.1016/j.freeradbiomed.2019.11.028 PMC6975167

[64] Bateman, H. G. & Jenkins, T. C. Method for extraction and separation by solid phase extraction of neutral lipid, free fatty acids, and polar lipid from mixed microbial cultures. *J. Agric. Food Chem.* **45**, 132–134 (1997).

[65] Wang, L. et al. Triglyceride-rich lipoprotein lipolysis releases neutral and oxidized FFAs that induce endothelial cell inflammation. *J. Lipid Res.* **50**, 204–213 (2009). 18812596 10.1194/jlr.M700505-JLR200 PMC2636918

[66] Zhao, P. et al. Artificial lipid droplets: novel effective biomaterials to protect cells against oxidative stress and lipotoxicity. *Nanomaterials (Basel)* **12**, 672 (2022). 35215001 10.3390/nano12040672 PMC8879118

[67] Li, H. et al. The landscape of cancer cell line metabolism. *Nat. Med.* **25**, 850–860 (2019). 31068703 10.1038/s41591-019-0404-8 PMC6629041

