# Supplementary material for: FSP1-mediated lipid droplet quality control prevents neutral lipid peroxidation and ferroptosis
Source: Nat Cell Biol. 2025 Oct 29;27(11):1902–13. doi: 10.1038/s41556-025-01790-y (PMC12611765; doi:10.1038/s41556-025-01790-y)
Supplement: Supplementary file 1 — Reporting Summary [file 41556_2025_1790_MOESM1_ESM.pdf]

nature portfolio

Corresponding author(s): James Olzmann and Mike Lange

Last updated by author(s): Aug 19, 2025

Reporting Summary

Nature Portfolio wishes to improve the reproducibility of the work that we publish. This form provides structure for consistency and transparency in reporting. For further information on Nature Portfolio policies, see our Editorial Policies and the Editorial Policy Checklist.

Statistics

For all statistical analyses, confirm that the following items are present in the figure legend, table legend, main text, or Methods section.

n/a	Confirmed	
☐	☒	The exact sample size (*n*) for each experimental group/condition, given as a discrete number and unit of measurement
☐	☒	A statement on whether measurements were taken from distinct samples or whether the same sample was measured repeatedly
☐	☒	The statistical test(s) used AND whether they are one- or two-sided<br>*Only common tests should be described solely by name; describe more complex techniques in the Methods section.*
☒	☐	A description of all covariates tested
☐	☒	A description of any assumptions or corrections, such as tests of normality and adjustment for multiple comparisons
☐	☒	A full description of the statistical parameters including central tendency (e.g. means) or other basic estimates (e.g. regression coefficient) AND variation (e.g. standard deviation) or associated estimates of uncertainty (e.g. confidence intervals)
☐	☒	For null hypothesis testing, the test statistic (e.g. *F*, *t*, *r*) with confidence intervals, effect sizes, degrees of freedom and *P* value noted<br>*Give P values as exact values whenever suitable.*
☒	☐	For Bayesian analysis, information on the choice of priors and Markov chain Monte Carlo settings
☒	☐	For hierarchical and complex designs, identification of the appropriate level for tests and full reporting of outcomes
☒	☐	Estimates of effect sizes (e.g. Cohen's *d*, Pearson's *r*), indicating how they were calculated

Our web collection on statistics for biologists contains articles on many of the points above.

Software and code

Policy information about availability of computer code

Data collection	IE Omics PMID: 28265968 https://innovativeomics.com/software/ie-omics/<br>DepMap Portal DOI: 10.1038/s41591-019-0404-8 https://depmap.org/portal/interactive/custom_analysis<br>Harmony 5.2 https://www.revvity.com/product/harmony-5-2-office-revvity-hh17000019?srsltid=AfmBOopeLtoI5DaneVedXzjFMZ2o2TGgj-cLr7wOTzq0kwgv7mWI-wfG
Data analysis	Graphpad Prism 10.2.2 (397) GraphPad Software https://www.graphpad.com/search/?searchquery=download<br>Harmony 5.2 https://www.revvity.com/product/harmony-5-2-office-revvity-hh17000019?srsltid=AfmBOopeLtoI5DaneVedXzjFMZ2o2TGgj-cLr7wOTzq0kwgv7mWI-wfG<br>ImageJ 1.54f doi:10.1038/nmeth.2089 https://imagej.net/downloads<br>LipoStar 2.0 DOI: 10.1021/acs.analchem.7b01259. https://www.moldiscovery.com/software/lipostar/<br>LPPtiger 2.0 DOI: 10.1038/s41598-017-15363-z https://github.com/SysMedOs/lpptiger<br>Metaboanalyst 5.0 DOI: 10.1093/nar/gkab382 https://metaboanalyst.ca/docs/About.xhtml<br>Proteowizard 3.0.9134 DOI: 10.1038/nbt.2377 https://proteowizard.sourceforge.io/download.html<br>Skyline PMID: 31984744 https://skyline.ms/project/home/software/skyline/begin.view

For manuscripts utilizing custom algorithms or software that are central to the research but not yet described in published literature, software must be made available to editors and reviewers. We strongly encourage code deposition in a community repository (e.g. GitHub). See the Nature Portfolio guidelines for submitting code & software for further information.

Data

Policy information about availability of data

All manuscripts must include a data availability statement. This statement should provide the following information, where applicable:

- Accession codes, unique identifiers, or web links for publicly available datasets
- A description of any restrictions on data availability
- For clinical datasets or third party data, please ensure that the statement adheres to our policy

Raw data and metadata will be reposited on MASSive in the GNPS ecosystem for download.

Research involving human participants, their data, or biological material

Policy information about studies with human participants or human data. See also policy information about sex, gender (identity/presentation), and sexual orientation and race, ethnicity and racism.

Reporting on sex and gender	N/A
Reporting on race, ethnicity, or other socially relevant groupings	N/A
Population characteristics	N/A
Recruitment	N/A
Ethics oversight	N/A

Note that full information on the approval of the study protocol must also be provided in the manuscript.

Field-specific reporting

Please select the one below that is the best fit for your research. If you are not sure, read the appropriate sections before making your selection.

☒ Life sciences ☐ Behavioural & social sciences ☐ Ecological, evolutionary & environmental sciences

For a reference copy of the document with all sections, see nature.com/documents/nr-reporting-summary-flat.pdf

Life sciences study design

All studies must disclose on these points even when the disclosure is negative.

Sample size	No sample size calculation was performed. A minimum sample size of n = 3 was applied in all assays. We deemed that this sample size is sufficient because we are identifying trends during exploratory research in highly controlled conditions and therefore are focusing on relationships with a high effect size.
Data exclusions	No data was excluded.
Replication	Functional testing of the anti-ferroptotic effect of various FSP1 constructs was repeated in technical duplicates on 3 different days and data was summed up and plotted together. For lipid analysis, no replication was performed due to the complexity of these analyses.
Randomization	Functional testing of the anti-ferroptotic function of various FSP1 constructs was not randomized. All mass spectrometry experiments were blinded and randomized during the data acquisition stage. Each sample was assigned a random number and sorted by decreasing values in excel, effectively randomizing samples to counteract methodological artifacts. Samples were unblinded after data analysis.
Blinding	Functional testing of the anti-ferroptotic function of various FSP1 constructs was not blinded. All lipid analyses were blinded, by assigning samples numbers before lipid extraction masking the sample identity.

Reporting for specific materials, systems and methods

We require information from authors about some types of materials, experimental systems and methods used in many studies. Here, indicate whether each material, system or method listed is relevant to your study. If you are not sure if a list item applies to your research, read the appropriate section before selecting a response.

Materials & experimental systems

n/a	Involved in the study
☐	☒ Antibodies
☐	☒ Eukaryotic cell lines
☒	☐ Palaeontology and archaeology
☒	☐ Animals and other organisms
☒	☐ Clinical data
☒	☐ Dual use research of concern
☒	☐ Plants

Methods

n/a	Involved in the study
☒	☐ ChIP-seq
☒	☐ Flow cytometry
☒	☐ MRI-based neuroimaging

Antibodies

Antibodies used	Goat Alexa Fluor 680 Anti-mouse IgG Thermo Fisher A28183<br>Mouse Anti-actin (monoclonal) Santa Cruz sc-47778<br>Mouse Anti-AMID (monoclonal) Santa Cruz sc-377120<br>Mouse Anti-GFP (monoclonal) Roche 11814460001
Validation	Anti-AMID antibody validated by blotting for AMID gene in wild type cells, AMID gene knockout and AMID re-expression in AMID gene knockout background. Other antibodies were used as is.

Eukaryotic cell lines

Policy information about cell lines and Sex and Gender in Research

Cell line source(s)	Cell lines were generated during the course of a previous study (DOI: 10.1038/s41586-019-1705-2) and were available in our laboratory.U-2OS parental cells, Caki-1 cells and HMC3 cells were obtained from the UC Berkeley Cell Culture Facility. Huh7 cells were a kind gift from Dr. Holly Ramage (University of Pennsylvania).
Authentication	Western blot was used to detect FSP1 and GFP constructs in all cell lines. Functional testing for successful FSP1 expression was performed by analyzing cell death kinetics in response to the Ferroptosis inducer RSL3 using time resolved fluorescence microscopy.
Mycoplasma contamination	Cell lines were tested for mycoplasma contamination in 4-6 months intervals throughout the duration of the study using the Mycoplasma PCR Detection Kit from Abcam (ab289834) and lines were always found to be free of mycoplasma contamination.
Commonly misidentified lines<br>(See ICLAC register)	N/A

Plants

Seed stocks	N/A
Novel plant genotypes	N/A
Authentication	N/A
