# Supplementary figures and images for: FSP1-mediated lipid droplet quality control prevents neutral lipid peroxidation and ferroptosis
Source: Nat Cell Biol. 2025 Oct 29;27(11):1902–13. doi: 10.1038/s41556-025-01790-y (PMC12611765; doi:10.1038/s41556-025-01790-y)

Extended Data Fig S1A

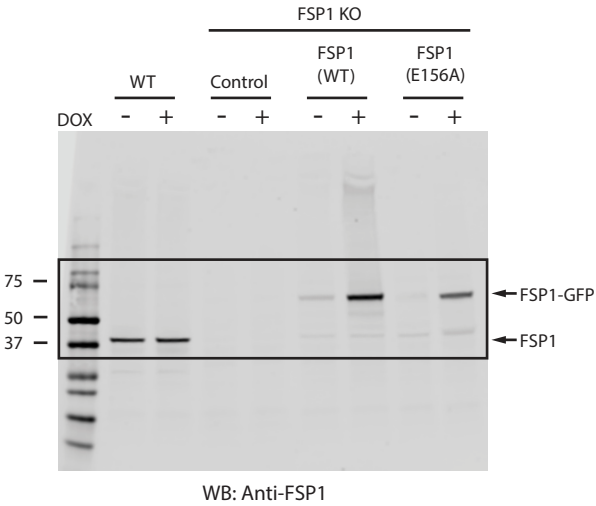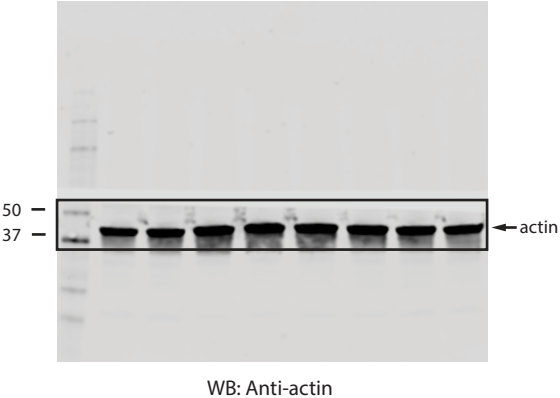

Supplement: Supplementary file 9 — Uncropped western blots. [file 41556_2025_1790_MOESM9_ESM.pdf]

Extended Data Fig S2A

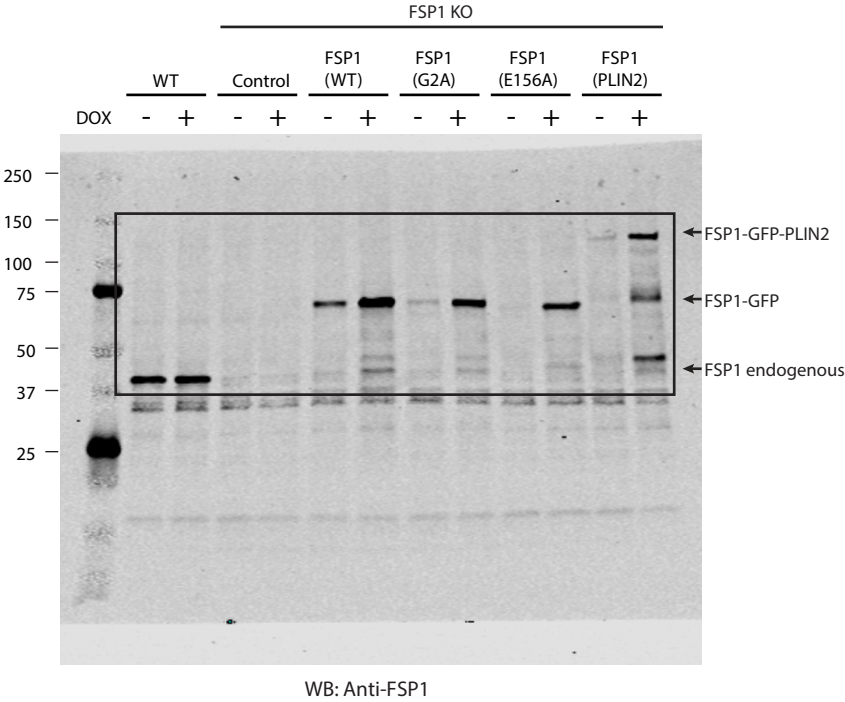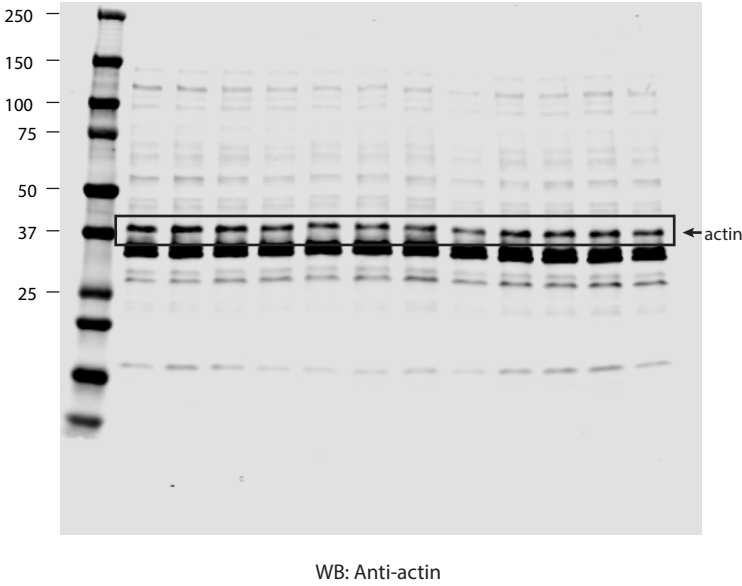

Extended Data Fig S2B

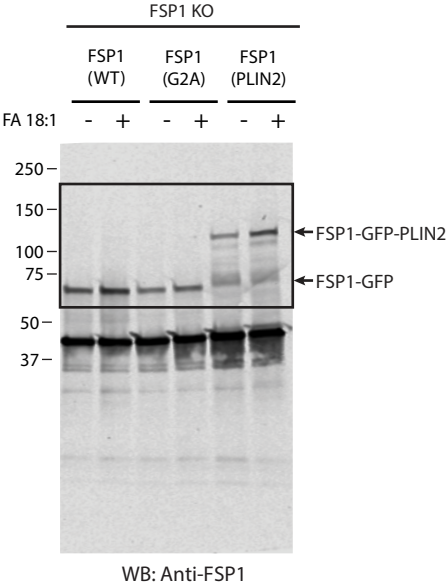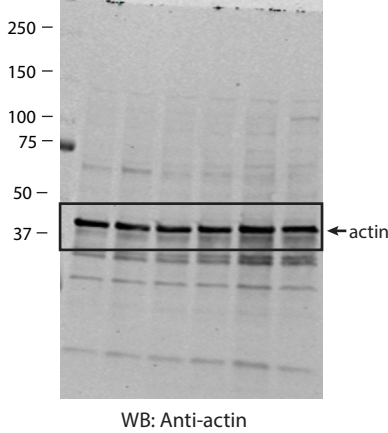

Supplement: Supplementary file 11 — Uncropped western blots. [file 41556_2025_1790_MOESM11_ESM.pdf]

Extended Data Fig S4A

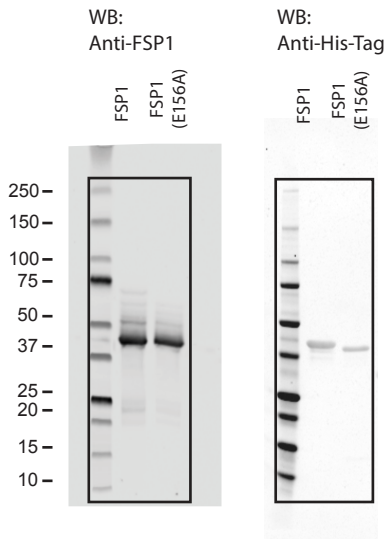

Extended Data Fig S4D

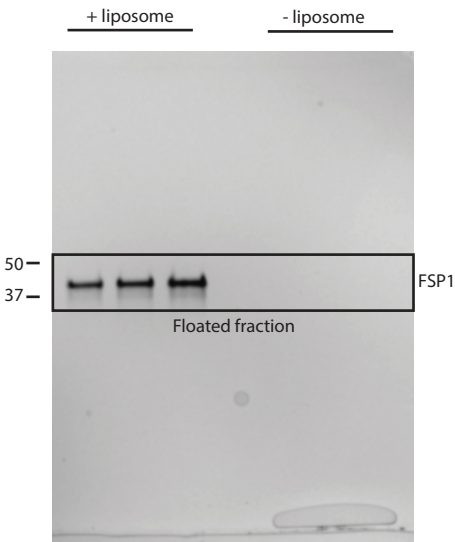

Extended Data Fig S4H

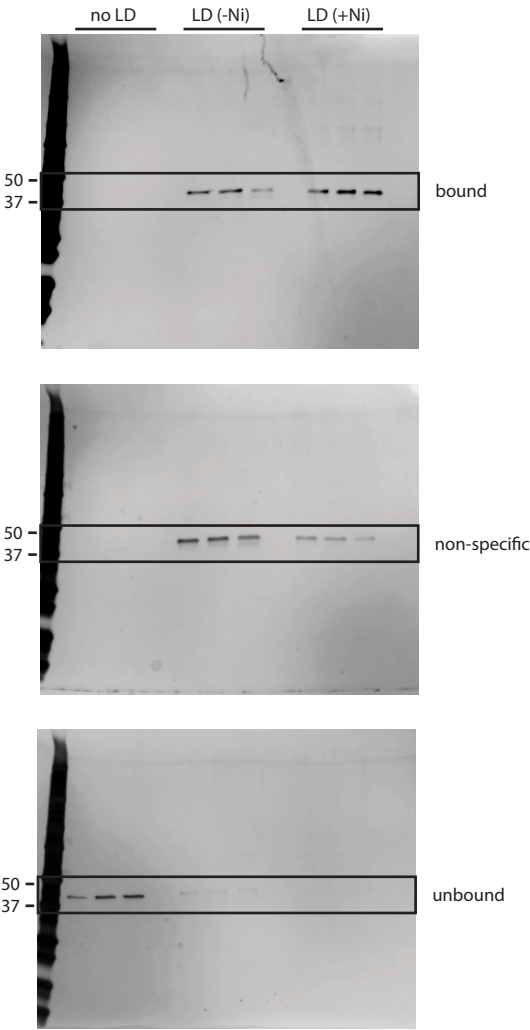

Supplement: Supplementary file 13 — Uncropped western blots. [file 41556_2025_1790_MOESM13_ESM.pdf]
